# A Meta-Population Model of Potential Foot-and-Mouth Disease Transmission, Clinical Manifestation, and Detection Within U.S. Beef Feedlots

**DOI:** 10.3389/fvets.2020.527558

**Published:** 2020-09-23

**Authors:** Aurelio H. Cabezas, Michael W. Sanderson, Victoriya V. Volkova

**Affiliations:** ^1^Department of Diagnostic Medicine and Pathobiology, College of Veterinary Medicine, Kansas State University, Manhattan, KS, United States; ^2^Center for Outcomes Research and Epidemiology, College of Veterinary Medicine, Kansas State University, Manhattan, KS, United States

**Keywords:** mathematical modeling, foot-and-mouth disease, transmission dynamics, meta-population, environmental transmission, waterborne transmission, beef feedlot, infectious disease dynamics

## Abstract

Foot-and-mouth disease (FMD) has not been reported in the U.S. since 1929. Recent outbreaks in previously FMD-free countries raise concerns about potential FMD introductions in the U.S. Mathematical modeling is the only tool for simulating infectious disease outbreaks in non-endemic territories. In the majority of prior studies, FMD virus (FMDv) transmission on-farm was modeled assuming homogenous animal mixing. This assumption is implausible for U.S. beef feedlots which are divided into multiple home-pens without contact between home-pens except fence line with contiguous home-pens and limited mixing in hospital pens. To project FMDv transmission and clinical manifestation in a feedlot, we developed a meta-population stochastic model reflecting the contact structure. Within a home-pen, the dynamics were represented assuming homogenous animal mixing by a modified SLIR (susceptible-latent-infectious-recovered) model with four additional compartments tracing cattle with subclinical or clinical FMD and infectious status. Virus transmission among home-pens occurred via cattle mixing in hospital-pen(s), cowboy pen rider movements between home-pens, airborne, and for contiguous home-pens fence-line and via shared water-troughs. We modeled feedlots with a one-time capacity of 4,000 (small), 12,000 (medium), and 24,000 (large) cattle. Common cattle demographics, feedlot layout, endemic infectious and non-infectious disease occurrence, and production management were reflected. Projected FMD-outbreak duration on a feedlot ranged from 49 to 82 days. Outbreak peak day (with maximum number of FMD clinical cattle) ranged from 24 (small) to 49 (large feedlot). Detection day was 4–12 post-FMD-introduction with projected 28, 9, or 4% of cattle already infected in a small, medium, or large feedlot, respectively. Depletion of susceptible cattle in a feedlot occurred by day 23–51 post-FMD-introduction. Parameter-value sensitivity analyses were performed for model outputs. Detection occurred sooner if there was a higher initial proportion of latent animals in the index home-pen. Shorter outbreaks were associated with a shorter latent period and higher bovine respiratory disease morbidity (impacting the in-hospital-pen cattle mixing occurrence). This first model of potential FMD dynamics on U.S. beef feedlots shows the importance of capturing within-feedlot cattle contact structure for projecting infectious disease dynamics. Our model provides a tool for evaluating FMD outbreak control strategies.

## Introduction

Foot-and-mouth disease (FMD) is a highly contagious disease affecting livestock and over a hundred wildlife species (1). Foot-and-mouth disease virus (FMDv) is of the genus *Apthovirus*, family *Picornaviridae*. There are seven antigenically distinct FMDv serotypes: A, O, C, SAT-1, SAT-2, SAT-3, and ASIA-1. Serotypes O and A are most widely distributed world-wide according to a recent review (2). In the Americas, major FMD outbreaks have not occurred since an outbreak in Paraguay in 2012, in which FMDv strains of serotype O predominated. An on-going program is aimed at eradicating FMD in South America by 2020 (3). The disease has not been reported in the U.S. since 1929 when southern California was affected (4). The last outbreak in North America occurred in 1952 in Saskatchewan, Canada (5). Economic impacts of an FMD outbreak in disease-free countries can be devastating due to export bans for susceptible animal species and their products, disease-associated animal losses, and outbreak control expenses. For example, the FMD outbreak in United Kingdom in 2001 resulted in the estimated overall costs over £8 billion ($15 billion) (6).

The U.S. beef industry is one of the largest in the world with over 30,000 feedlots, primarily concentrated in the Central U.S. (7). Almost 50% of the national fed cattle inventory are in large commercial feedlots, each with the on-time capacity ≥24,000 head of cattle. Approximately 1,160 million kilograms of beef are exported by the U.S. producers each year (8). Response by the world animal-health community to an FMD outbreak in the U.S. would likely involve a ban on beef exports. Schroeder et al. (9) estimate that an FMD outbreak in the U.S. could result in $188 billion overall costs without emergency vaccination and $56 billion with high-capacity emergency vaccination in the Midwest. Pendell et al. (10) estimated $16–140 billion costs for an outbreak if FMDv would be released from a high-security laboratory facility in the Midwest. Others estimated a decrease in farm income of $14 billion, ~6% of the national gross farm income, in the U.S. for an outbreak assuming the outbreak characteristics were similar to the UK 2001 outbreak (11).

For long-term FMD-free countries, such as the U.S., mathematical modeling is the only tool for projecting dynamics of a potential FMD outbreak and evaluating control strategies. Previous modeling studies of FMDv transmission and control in the U.S. focused on projecting the impact on the outbreak of the virus transmission dynamics between farms. In the models, individual farms were considered as FMD positive or negative (12–17). A similar assumption has been made in models of FMD outbreaks in territories other than the U.S. (18–25).

In a U.S. beef cattle feedlot, the cattle are compartmentalized in multiple home-pens (e.g., 200 head per home-pen). The home-pen subpopulations contact via multiple routes conducive to contagious agent transmission, forming the meta-population of cattle in the feedlot. There is a multi-route, complex, and heterogeneous in time and space contact structure among the home-pen subpopulations. The relevant contact routes include mixing of some cattle from different home-pens during short stays in hospital-pens, fence-line contact for contiguous home-pens, waterborne contact for contiguous home-pens sharing water-troughs, environmental due to the care-givers moving between the home-pens located in the same home-pen row (the rows are separated by feed-delivery alleys and drover alleys), and airborne across the feedlot. Thus, an assumption of a contagious virus transmission via an instantaneous and homogeneous mixing of all cattle present on a feedlot is implausible. Projecting the transmission among the home-pen subpopulations necessitates a more explicit model of the contact structure. Reflecting the meta-population contact structure when modeling infectious agent transmission is necessary because the agent temporal dynamics and likelihood of persistence in a meta-population are different from in a homogenously mixing population (26–28). Three teams have modeled within-farm FMDv transmission in cattle (14, 29, 30). However, the animal contact structure, demographics, and production management represented were dissimilar to those in U.S. beef feedlots. One study (31) modeled within-farm FMDv transmission in swine. Models of potential FMDv transmission dynamics in the cattle meta-populations on U.S. beef feedlots have not been reported.

The aim of this study was to develop a mathematical model of potential FMDv transmission, infection, and FMD clinical manifestation dynamics in U.S. beef feedlots, reflecting the animal meta-population contact structure, animal demographics, and contemporary production management. The model was developed as a stochastic meta-population model. In the model, FMDv transmission within a home-pen occurred via homogenous cattle mixing. Relevant contacts among the home-pen subpopulations occurred via cattle mixing in hospital-pen(s), and through fence-lines, shared water-troughs, environment due to care-giver movements between the home-pens in a row, and airborne. The model reflected commercial U.S. beef cattle feedlot demographics and production management, including the incidence and control approaches to endemic infectious diseases and non-infectious diseases. We used the model to project FMDv infection dynamics and clinical manifestation in the absence of control measures on feedlots of several sizes and layouts typical for the U.S. We analyzed the model outputs to describe the projected outbreak characteristics. To our knowledge, this is the first model of potential FMD dynamics on commercial U.S. beef feedlots.

## Materials and Methods

### Host Population and Feedlot Size and Layout Cases Modeled

**Figure 1:**
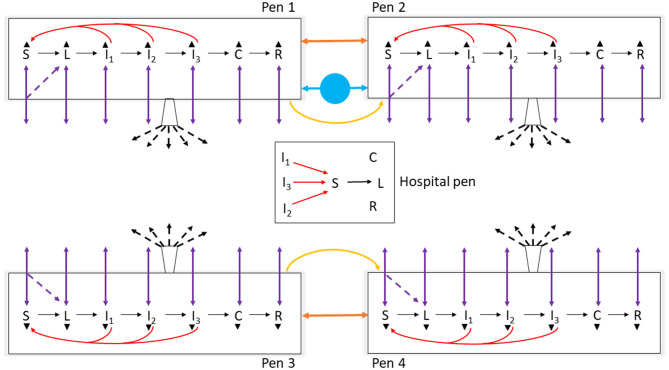
Schematic diagram of the model of foot-and-mouth disease (FMD) virus transmission and FMD clinical manifestation dynamics in cattle within home-pens and among home-pens in a beef cattle feedlot. S, susceptible; L, latent; I_1_, subclinical infectious; I_2_, subclinical infectious; I_3_, clinical infectious; C, clinical but no longer infectious; and R, non-infectious clinically recovered. The black solid arrows show the home-pen subpopulation progression through the infection and disease stages. The red solid arrows show the virus transmission via direct contact between infectious to susceptible cattle in the home-pens and hospital-pen. The purple solid arrows show the animal movements from home-pens to the hospital-pen and back to the home-pens, and the purple dotted arrows show the possibility that susceptible animals moved acquired infection in the hospital-pen and returned as latent to the home-pens. The orange solid arrows show the virus transmission via animal direct contact fence-line. The yellow solid arrows show the virus contaminated material transmitted by pen-riders. The blue circle with solid arrows shows the virus transmission via contaminated water-troughs shared by home-pens. The buckets with black dotted arrows represent the airborne virus transmission. The black triangles represent animal mortality in each of the infection and disease stages.

The model reflected the following assumptions. Beef finishing cattle in an open-air feedlot was the target population. No other FMD-susceptible animal species were included on the feedlot or in the surroundings. The cattle were not vaccinated against FMD. Cattle were housed 200 per home-pen, with 22 m^2^ floor space per animal. Cattle morbidity due to production diseases including endemic infectious diseases and non-infectious diseases, e.g., bovine respiratory disease (BRD) and lameness, determined the rate of pulling cattle from the home-pens to hospital-pen(s). Cattle mortality rates due to the production diseases and clinical FMD were incorporated. The model parameter definitions and values are listed in Table 1. We simulated the feedlot cattle meta-population as closed, with no cattle introduced or leaving the feedlot after FMD latent animals were introduced in the index home-pen. Five hypothetical feedlot size and layout cases were modeled: a small-size feedlot with 4,000 cattle in 20 home-pens in four rows and one hospital-pen (FS1); a medium-size feedlot with 12,000 cattle in 60 home-pens in eight rows and one hospital-pen (FM1); a medium-size feedlot with 12,000 cattle in 60 home-pens in eight rows and two hospital-pens, (FM2); a large-size feedlot with 24,000 cattle in 120 home-pens and two hospital-pens, the feedlot includes two sections each with eight home-pen rows and one hospital-pen (FL1); and a large-size feedlot with 24,000 cattle in 120 home-pens and four hospital-pens, the feedlot includes two sections each with eight home-pen rows and two hospital-pens (FL2). See Figure 1 for a schematic diagram of the model. The feedlot layouts are detailed in Supplementary Figure 1.

**Table 1:** Definitions and values of parameters used in modeling potential foot-and-mouth disease transmission, infection, and clinical manifestation dynamics on U.S. beef cattle feedlots.

**Parameter**	**Definition (units)**	**Mean value and distribution**	**References^**a**^**
**WITHIN A HOME-PEN**			
*lat_initial*	Initial proportion of latent cattle in the index-pen	0.05, Vector (0.005, 0.105, 0.020)	Assumed
*β_*wp*_*	Beta transmission parameter for virus transmission via direct animal contact in a home-pen (animal^−1^ day^−1^)	0.026, Triangular (0.020, 0.026, 0.031)	Derived from Chis Ster et al. (30)
*lat*	Duration of latent period (days)	3.2, Weibull (α 1.782, β 3.974)	(32)
*sub*	Duration of subclinical period (days)	2.0, Gamma (α 1.222, β 1.672)	(32)
*inf*	Duration of infectious period (days)	4.0, Gamma (α 3.969, β 1.107)	(32)
*cli*	Duration of clinical period (days)	7.5, Fixed	(33)
*cliinf*	Duration of clinical infectious period (days)	(inf-sub) in each model simulation	
*clinon_inf*	Duration of clinical non-infectious period (days)	(cli-clininf) in each model simulation	
δ	Rate of progression to subclinical infectious 1 status (day^−1^)	1/lat	
θ	Rate of progression to subclinical infectious 2 status (day^−1^)	1/(sub/2)	
ε	Rate of progression to clinical infectious status (day^−1^)	1/(sub/2)	
γ	Rate of recovery from being infectious (day^−1^)	1/cliinf	
τ	Rate of recovery from clinical disease after recovering from being infectious (day^−1^)	1/clinon_inf	
υ	Proportion of home-pens with cattle just placed in the feedlot (dmnl)	0.20	Feedlot expert opinion
π	Morbidity rate for bovine respiratory disease (BRD) during the first 30 days since cattle placement in the feedlot	0.162, Vector (0.050, 0.300, 0.050)	(34)
ρ	Morbidity rate for other production diseases during the 200 days since cattle placement in the feedlot	0.1280, fixed	(34)
*brdtrt*	Probability for an animal with BRD to be pulled to a hospital-pen for treatment during the disease course (dmnl)	0.8750, fixed	(34)
*endtrt*	Probability for an animal with other than BRD production diseases to be pulled to a hospital-pen for treatment during the disease course (dmnl)	0.6908, fixed	(34)
φ_*t* = 1to 30_	Per-animal pull rate from a home-pen to hospital-pen due to BRD and other production diseases during the first 30 days since cattle placement in the feedlot (day^−1^)	0.0052	Calculated, $\left(\frac{\pi *brdtrt}{30}\right)+\left(\frac{\rho *endtrt}{200}\right)$
φ_*t* = 31to 200_	Per-animal pull rate from a home-pen to hospital-pen due to production diseases between the days 31 and 200 since cattle placement in the feedlot (day^−1^)	0.0004	Calculated, $\frac{\rho *endtrt}{200}$
ς	Per-animal pull rate from a home-pen to hospital-pen due to clinical FMD (day^−1^)	0.02800	FMD expert opinion
μ	Mortality rate for animals with BRD and other production diseases (endemic infectious diseases and noninfectious diseases) (day^−1^)	Triangular (0.01, 0.03, 0.05)	(34)
ψ	Mortality rate for animals with clinical FMD (day^−1^)	Triangular (0, 0.005, 0.010)	FMD expert opinion
**BETWEEN HOME-PENS**			
**In hospital-pen(s)**			
*β_*hp*_*	Beta transmission parameter for virus transmission via direct animal contact in a hospital-pen (animal^−1^ day^−1^)	Same as *β_*wp*_*	Derived from Chis Ster et al. (30)
**Fence-line**			
*β_*bp*_*	Beta transmission parameter for virus transmission via fence-line direct animal contact (animal^−1^ day^−1^)	*β_*wp*_*/4	Assumed [*β_*wp*_* derived from Chis Ster et al. (30)]
**Environmental by pen-riders**			
*uri*	Urine volume produced by an animal (L/day)	Uniform (8.8, 22.0)	(35)
*sal*	Saliva volume produced by an animal (L/day)	Uniform (98, 190)	(35)
*fec*	Volume of feces produced by an animal (kg/day)	Uniform (14, 45)	(35)
*uriv*	Virus quantity shed in urine [plaque forming units (PFU)/mL] by an animal in the FMD clinical high infectious status	Uniform (10^2.5^, 10^5.5^)	(35)
*salv*	Virus quantity shed in saliva (PFU/mL) by an animal in the FMD clinical high infectious status	Uniform (10^6^, 10^8^)	(35)
*fecv*	Virus quantity shed in feces (PFU/mL) by an animal in the FMD clinical high infectious status	Uniform (10^2^, 10^4.1^)	(35)
*fsal_env*	Proportion of the cattle daily saliva volume deposited into the home-pen environment (dmnl)	0.3, Vector (0.1, 0.5, 0.1)	Assumed
*fsal_env_floor*	Proportion of *fsal* that lands on the floor (dmnl)	0.33	Assumed
*vir_dec_env*	Virus decay rate in the home-pen floor environment (day^−1^)	0.28, Fixed	(36)
σ	Amount of the home-pen floor materials moved daily to the next home-pen in the row by pen-riders (g/day) (300 g per pen-rider round, two rounds per day)	600, Fixed	Assumed plausible amount carried on horse hooves between pens
*w_pen*	Width of a home-pen (m)	61.0, Fixed	Typical industry value
*l_pen*	Length of a home-pen (m)	75.2, Fixed	Typical industry value
*d_pen*	Depth of a home-pen floor top contaminated with the animal fresh secretions and excretions (m)	0.02, Vector (0.02, 0.05, 0.03)	Expert opinion, typical pen surface loosened by hoof action
*min_oral*	Minimum infective dose of FMDv via oral exposure in cattle (PFU/mL)	10^6^, Fixed	(37)
**Via shared water-troughs**			
*fsal_env_w*	Proportion of *fsal* that lands in the water-trough (dmnl)	(1-*fsal_env_floor*)	Assumed
*vir_dec_w*	Virus decay rate in water (day^−1^)	0.12, Fixed	(36)
*vol_watert*	Volume of the water trough shared between two home-pens (L)	6,000, Fixed	Expert opinion, typical tank size to provide sufficient water reservoir for cattle needs
*min_oral*	Minimum infective dose of FMDv via oral exposure in cattle (PFU/mL)	10^6^, Fixed	(37)
**Airborne**			
α	Power of the exponential function of decay in the airborne transmission with increasing distance between home-pen centroids (dmnl)	−3.5, Fixed	(24)
	Proportion of clinical infectious cattle in a home-pen *k*	Modeled	
*d_*i*_*_, **k**_	Scaled distance between centroids of a home-pen *i* and home-pen *k* (*k* is any other home-pen than *i*) (dmnl)	1.0–22.4, Fixed	Euclidean distance between each two home-pen centroids scaled by the shortest Euclidian distance between two home-pen centroids in the feedlot

a *In the reference column: “Assumed” refers to parameter values assigned based on our knowledge/judgement. “Derived from [x]” refers to values that we estimated based on data in the cited references. “[x]” is the reference from which the value was adopted directly. “Expert opinion” refers to values obtained via personal communication with experts in the epidemiology of FMD, and in the feedlot industry*. *dmnl, indicates the value does not have a unit of measure*. *PFU, plaque forming units*.

**Table 2:** Characteristics of projected foot-and-mouth disease outbreaks on a U.S. beef cattle feedlot.

**Feedlot**	**Number of home-pens**	**Total number of cattle**	**Number of sections of home-pens**	**Number of hospital-pens**	**Outbreak duration^**a**^, days (10th, 50th, and 90th percentiles of *n* = 2,000 simulated outbreaks)**	**Outbreak peak day with highest number of cattle with clinical FMD (10th, 50th, and 90th percentiles of *n* = 2,000 simulated outbreaks)**	**Day of FMD outbreak detection**^****b****^ **based on detection threshold of 3, 5, and 10% clinical animals in the index home-pen (10th, 50th, and 90th percentiles of** ***n*** **=** **2,000 simulated outbreaks)**		
							**3%**	**5%**	**10%**
FS1	20	4,000	1	1	39, 49, 59	21, 23, 27	4, 6, 9	5, 6, 10	6, 7, 12
FM1	60	12,000	1	1	46, 58, 69	25, 28, 33	4, 6, 9	5, 6, 10	6, 7, 12
FM2	60	12,000	1	2	61, 74, 89	26, 31, 43	4, 6, 9	5, 6, 10	6, 7, 12
FL1	120	24,000	2	2	60, 73, 86	33, 41, 48	4, 6, 9	5, 6, 10	6, 7, 12
FL2	120	24,000	2	4	68, 82, 95	42, 49, 57	4, 6, 9	5, 6, 10	6, 7, 12

a * Outbreak duration was defined as the number of days since introduction of FMD latent cattle in the index home-pen on the feedlot until the last animal infected within the feedlot proceeded from the clinical high infectious stage to the clinical non-infectious stage*.

b *Outbreak detection was assumed to occur via routine visual surveillance of cattle health by the pen-riders, on the day when proportion of cattle with clinical FMD in the index home-pen reached 3, 5, and 10%^∧^. This detection threshold was assumed to be independent of the feedlot size (cattle head count)*.

### Model Formulation

Two levels of FMDv transmission inside the feedlot cattle meta-population were modeled: within each home-pen (1 route of transmission: direct cattle contact) and between home-pens (5 routes of transmission detailed below).

#### FMD Infection and Clinical Manifestation Dynamics in a Home-Pen

**Figure 2:**
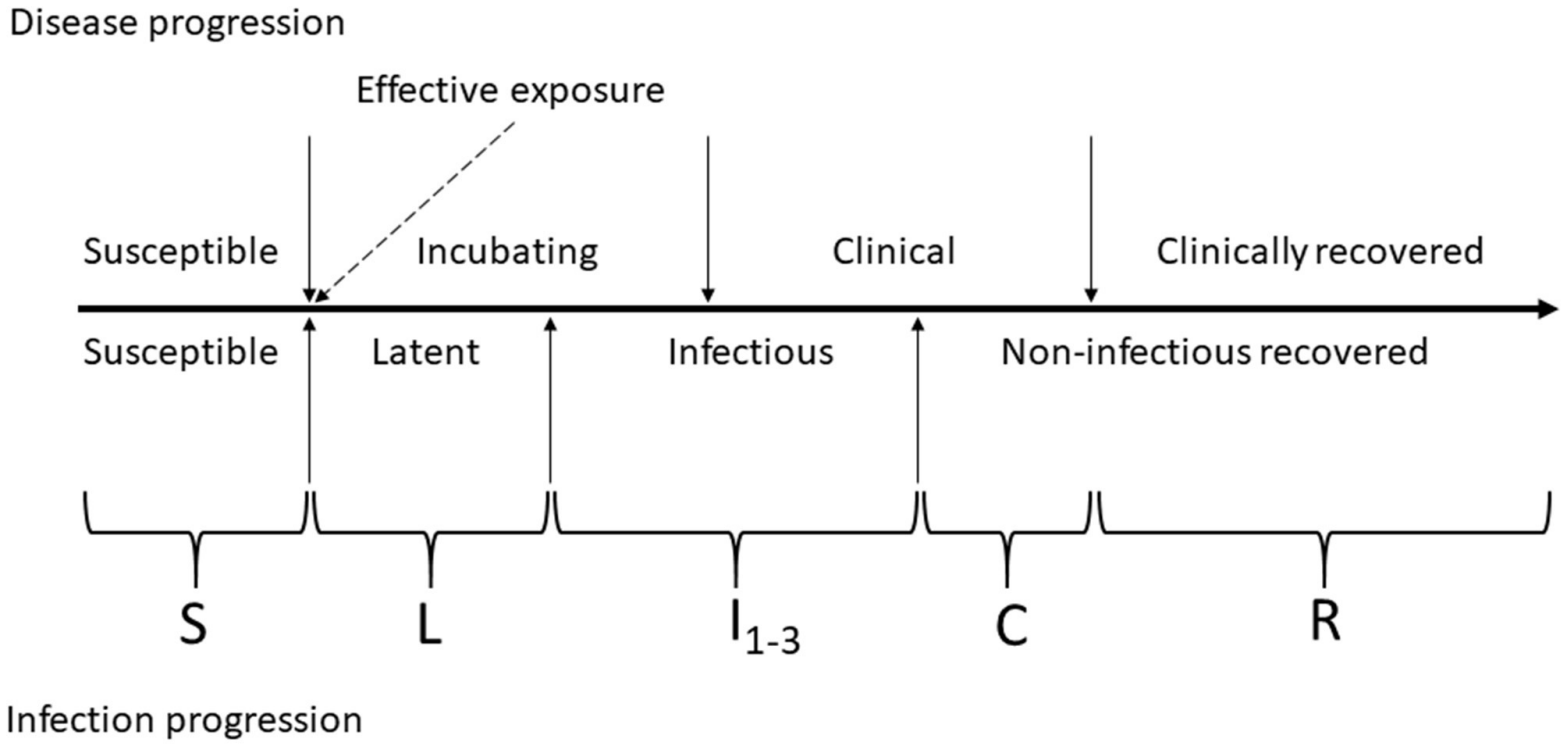
Schematic of foot-and-mouth disease (FMD) infection and clinical disease progression in individual cattle. The compartments of the modified SLIR model of FMD dynamics are indicated by letters: S, susceptible; L, latent; I_1_, subclinical infectious; I_2_, subclinical–infectious; I_3_, clinical infectious; C, clinical but no longer infectious; and R, non-infectious clinically recovered.

The FMD infection and clinical disease dynamics in each home-pen were modeled using a modified SLIR (susceptible-latent-infectious-recovered) model. The model was modified to add four compartments for tracing the numbers of cattle that were subclinical infectious 1 (I_1_ animals), subclinical infectious 2 (I_2_ animals), clinical infectious (I_3_ animals), and clinical non-infectious (C). The two subclinical categories were of equal duration and infectiousness but were included to allow for future parameterization of variability in infectiousness between stages. A schematic of the infection and clinical disease progression stages in individual cattle and how those were reflected in the model compartments is provided in Figure 2. Cattle started in the susceptible compartment (S) (Equation 1). Susceptible cattle were infected via direct contact with infectious home-pen-mates at a rate reflecting homogenous cattle mixing and density-dependent transmission within the home-pen (the transmission parameter β_*wp*_, Equation 1) or due to between-home pen FMDv transmission (detailed below) and moved into the latent compartment (L) (Equations 1, 2). The cattle then moved into a subclinical infectious compartment (I_1_) at a rate 1/δ (Equations 2, 3), proceeded into a subclinical infectious compartment (I_2_) at a rate 1/θ (Equations 3, 4), then into a clinical infectious compartment (I_3_) at a rate 1/ε (Equations 4, 5), and then into a clinical non-infectious compartment (C) at a rate 1/γ (Equations 5, 6) where they were still manifesting clinical disease but no longer shed the virus. Finally, the cattle proceeded into a non-clinical non-infectious recovered compartment (R) at a rate 1/τ (Equations 6, 7). Cattle mortality (i.e., culling) due to endemic infectious diseases and non-infectious diseases occurred at a rate μ in all the compartments (Equations 1–7). Cattle mortality (i.e., culling) due to clinical FMD in the compartments I_3_ and C occurred at a rate ψ (Equations 5, 6). Definitions and values of the model parameters are given in Table 1.

The modified SLIR model of FMD infection and clinical manifestation dynamics in cattle in a home-pen on a beef feedlot

The modeled home-pen is denoted *i. j* is the contiguous home-pen preceding *i* in the home-pen row. *h* is the contiguous home-pen following *i* in the home-pen row. *k* is any other home-pen than *i*. *n* is the number of home-pens in the feedlot. If the feedlot had more than one hospital-pen, cattle were always pulled to the hospital-pen nearest to their home-pen for either a production disease or clinical FMD treatment. The nearest hospital-pen, or the only hospital-pen if there was one on the feedlot, is denoted *l*. The other parameters are defined in the following sections on the FMDv transmission between home-pens. The time step was 1 day, *dt* = 1 (all the rates in the equations including those with the values sampled from Binomial distributions are daily rates).

Susceptible:

(1) dSdt=-βwpS(I1+I2+I3)-φS-Bin(φ(t-1)S(t-1), p_inf_hpl(t-1))-{Sβbp(I1+I2+I3)j; j present0; otherwise}-{Sβbp(I1+I2+I3)h; h present0; otherwise}-{Bin(S, 0.5); j present, shares water-trough with i, and FMDv load in 1 L of the water ≥ID50 per oral0; otherwise}-{Bin(S, 0.5); h present, shares water-trough with i, and FMDv load in 1 L of the water ≥ID50 per oral0; otherwise}-{Bin[(FMDv_floorj×σID50 per oral), 0.5]; j present and(FMDv_floorj×σID50 per oral)≤S0; otherwise}-{Bin(S, p_airi); ∑k=1nI3≥00; otherwise}-μS

Latent:

(2) dLdt=βwpS(I1+I2+I3)-φL+Bin(φ(t-1)S(t-1), p_inf_hpl(t-1))+{Sβbp(I1+I2+I3)j; j present0; otherwise}+{Sβbp(I1+I2+I3)h; h present0; otherwise}+{Bin(S, 0.5); j present, shares water-trough with i, and FMDv load in 1 L of the water ≥ID50 per oral0; otherwise}+{Bin(S, 0.5); h present, shares water-trough with i, and FMDv load in 1 L of the water ≥ID50 per oral0; otherwise}+{Bin[(FMDv_floorj×σID50 per oral), 0.5]; j present and(FMDv_floorj×σID50 per oral)≤S0; otherwise}+{Bin(S, p_airi); ∑k=1nI3≥00; otherwise}-δL-μL

Subclinical infectious 1:

(3) $$\begin{array}{l} \frac{\text{d}{\text{I}}_{\text{1}}}{\text{dt}}=\delta \text{L}-\theta {\text{I}}_{\text{1}}-\varphi {\text{I}}_{\text{1}}+\varphi_{\left(t-1\right)}{\text{I}}_{{\text{1}}_{\left(t-1\right)}}-\mu {\text{I}}_{1} \end{array}$$

Subclinical infectious 2:

(4) $$\begin{array}{l} \frac{\text{d}{\text{I}}_{\text{2}}}{\text{dt}}=\theta {\text{I}}_{\text{1}}-\varepsilon {\text{I}}_{\text{2}}-\varphi {\text{I}}_{\text{2}}+\varphi_{\left(t-1\right)}{\text{I}}_{{\text{2}}_{\left(t-1\right)}}-\mu {\text{I}}_{\text{2}} \end{array}$$

Clinical infectious:

(5) $$\begin{array}{l} \frac{\text{d}{\text{I}}_{\text{3}}}{\text{dt}}=\varepsilon {\text{I}}_{\text{2}}-\gamma {\text{I}}_{\text{3}}-\left(\varphi +\varsigma \right){\text{I}}_{\text{3}}+\left(\varphi_{\left(t-1\right)}+\varsigma \right){\text{I}}_{{\text{3}}_{\left(t-1\right)}}-\left(\mu +\psi \right){\text{I}}_{\text{3}} \end{array}$$

Clinical non-infectious:

(6) $$\begin{array}{l} \frac{\text{dC}}{\text{dt}}=\gamma {\text{I}}_{\text{3}}-\tau \text{C}-\left(\varphi +\varsigma \right)\text{C}+\left(\varphi_{\left(t-1\right)}+\varsigma \right){\text{C}}_{\left(t-1\right)}-\left(\mu +\psi \right)\text{C} \end{array}$$

Recovered:

(7) $$\begin{array}{l} \frac{\text{dR}}{\text{dt}}=\tau \text{C}-\varphi \text{R}+\varphi_{\left(t-1\right)}{\text{R}}_{\left(t-1\right)}-\mu \text{R} \end{array}$$

#### FMDv Transmission Between Home-Pens

Transmission of FMDv between the home-pen subpopulations occurred via five routes: direct contact of cattle from different home-pens in hospital-pen(s) when they were pulled from the home-pen for treatment in the hospital, fence-line direct contact of cattle from contiguous home-pens, environmental contact through pen-riders moving between home-pens in the same home-pen row (only from a preceding to the next home-pen in the row), waterborne between contiguous home-pens that shared a water-trough, and airborne.

i. *Transmission via direct contact of cattle in hospital-pen(s)*

An S-L (Susceptible-Latent) model was implemented in each hospital-pen *l*. The susceptible and infectious (I_1_-I_3_) cattle originated from the home-pens when morbid cattle were sent to this hospital-pen. The new latents infected in the hospital-pen and remaining susceptibles (as well as the prior infectious, prior clinical non-infectious, and prior recovered pulled to the hospital-pen) returned to their home-pens the next day (Equations 1–7). Recall that cattle from a home-pen were always pulled to the nearest hospital-pen, except in the FS1 and FM1 feedlots where all cattle were pulled to the single hospital-pen.

From a home-pen, a number of cattle were daily pulled to the hospital-pen due to production diseases—endemic infectious and non-infectious diseases—and returned next day; the per-animal daily pull probability (φ) was equal for all cattle irrespective of their FMD status. This probability was a product of the expected production disease morbidity and the probability to be pulled to the hospital-pen for treatment depending on the disease. For cattle in a home-pen, the expected production disease morbidity was the sum of the bovine respiratory disease (BRD) daily morbidity (π) during the first 30 days after placement onto the feedlot, and the aggregated daily morbidity for all other production diseases (ρ), such as lameness and digestive conditions during the entire 200-days period in the feedlot. The probability of cattle with BRD to be pulled to the hospital-pen for treatment was *brdtrt* and with the other diseases it was *endtrt*. The total per-animal daily probability to be pulled due to the production diseases from a home-pen to the hospital-pen was φ = π * *brdtrt* + ρ * *endtrt*. In a feedlot, cattle are placed in individual home-pens, i.e., placed “on-feed,” at different times; all cattle are placed in a given home-pen simultaneously. At the start of the model simulations, a fraction (υ) of the home-pens were assumed to just have been placed (day 1 in the feedlot); the home-pens were assigned randomly using a random number generator. The rest of the home-pens were assumed to have been placed >30 days prior. There was also a per-animal daily probability (ς) to be pulled to the hospital-pen for cattle with clinical FMD.

In a hospital-pen, there was homogenous mixing of the cattle pulled from different home-pens that day. The susceptible cattle were infected via direct contact with infectious cattle (I_1_-I_3_) at a rate reflecting the homogenous mixing and density-dependent transmission (as in the home-pens), and with the same transmission parameter value, β_*hp*_in the hospital-pen(s) = β_*wp*_.

S-L Susceptible-Latent model of FMD infection dynamics in a hospital-pen l

$$\begin{array}{l} \frac{\text{d}{\text{S}}_{hpl}}{\text{dt}}=\overset{m}{\underset{i=1}{\sum }}\varphi {\text{S}}_{i}-\beta_{hp}\overset{m}{\underset{i=1}{\sum }}\varphi {\text{S}}_{i}\left[\overset{m}{\underset{i=1}{\sum }}\varphi {\text{I}}_{{\text{1}}_{i}}+\overset{m}{\underset{i=1}{\sum }}\varphi {\text{I}}_{{\text{2}}_{i}}+\overset{m}{\underset{i=1}{\sum }}\left(\varphi +\varsigma \right){\text{I}}_{{\text{3}}_{i}}\right] \end{array}$$

$$\begin{array}{l} \frac{\text{d}{\text{L}}_{hpl}}{\text{dt}}=\beta_{hp}\overset{m}{\underset{i=1}{\sum }}\varphi {\text{S}}_{i}\left[\overset{m}{\underset{i=1}{\sum }}\varphi {\text{I}}_{{\text{1}}_{i}}+\overset{m}{\underset{i=1}{\sum }}\varphi {\text{I}}_{{\text{2}}_{i}}+\overset{m}{\underset{i=1}{\sum }}\left(\varphi +\varsigma \right){\text{I}}_{{\text{3}}_{i}}\right] \end{array}$$

Where *i* is a home-pen, *m* is the number of home-pens from which cattle are pulled to the hospital-pen *l*, and φ and ς are defined in the preceding paragraph. All the parameters are also defined in Table 1. *m* = *n* if the feedlot had one hospital-pen. The probability for a susceptible animal pulled to the hospital-pen *l* to be infected by FMD in the hospital-pen that day was:

$$\begin{array}{l} p\text{\_inf\_h}{\text{p}}_{l}=\frac{\beta_{hp}\overset{m}{\underset{i=1}{\sum }}\varphi {\text{S}}_{i}\left[\overset{m}{\underset{i=1}{\sum }}\varphi {\text{I}}_{{\text{1}}_{i}}+\overset{m}{\underset{i=1}{\sum }}\varphi {\text{I}}_{{\text{2}}_{i}}+\overset{m}{\underset{i=1}{\sum }}\left(\varphi +\varsigma \right){\text{I}}_{{\text{3}}_{i}}\right]}{\overset{m}{\underset{i=1}{\sum }}\varphi {\text{S}}_{i}} \end{array}$$

The number of latent cattle returning to a home-pen *i* that were pulled a day earlier to the hospital-pen *l* while still susceptible and infected by FMD in the hospital-pen was

$$\begin{array}{l} Bin\left(\varphi_{\left(t-1\right)}{\text{S}}_{i_{\left(t-1\right)}},\;p\text{\_inf\_h}{\text{p}}_{l_{\left(t-1\right)}}\right). \end{array}$$

ii *Fence-line transmission via direct contact of cattle from contiguous home-pens*

A fence-line contact between cattle from neighboring home-pens is typical on U.S. feedlots. Home-pens are separated by fences, which do not prevent animal nose-to-nose contact. The fence-line FMDv transmission between each two contiguous home-pens was modeled assuming a homogenous animal mixing and density-dependent transmission along the fence (Equations 1, 2). The effective contact rate fence-line was assumed to be 25% of that within the home-pens, β_*bp*_ = β_*wp*_ × 0.25. Definitions and values of the parameters are given in Table 1. The number of cattle infected on a given day by FMD in a home-pen *i* via the fence-line transmission from a contiguous home-pen *j* (or home-pen *h* on the other side of *i*) was S_*i*_β_*bp*_(I_1_+I_2_+I_3)_*j*(or *h*)__.

iii *Environmental transmission due to pen-riders moving between home-pens*

Beef feedlots in the U.S. employ personnel to visually monitor cattle health as an observational disease surveillance method; they are known as pen-riders, pen-checkers, or cowboys and move between the home-pens on foot or on horses. The home-pen floor materials attached to the pen-rider boots or horse hooves could serve as a fomite for FMDv transmission. Such environmental virus transmission between each two contiguous home-pens sequentially visited by a pen-rider in the same home-pen row was modeled (see Supplementary Figures 1A–E for the feedlot layouts modeled). A possibility of such environmental transmission between the home-pen rows separated by feed-delivery or drover alleys was not modeled, assuming that majority of the floor materials picked up by a pen-rider in a home-pen are deposited in the next visited home-pen.

In the originating home-pen *j* we considered:

- The daily volumes of cattle secretions (saliva) and excretions (urine and feces) in which FMDv can be shed,
- The fractions of the secretions deposited into the home-pen environment and then on the floor (the excretions were assumed to be entirely deposited on the floor),
- The viral quantities shed per unit volume of each of the secretions and excretions by an animal in the clinical high infectious FMD stage (I_3_),
- The floor size and floor top depth that can be contaminated by the secretions and excretions, and
- The daily viral decay in the floor materials were reflected to model the remaining viral load in the floor materials.

We assumed that only secretions and excretions from the I_3_ cattle contributed to this transmission route. Each I_3_ animal daily excreted *uri* urine and *fec* fecal volumes, and secreted *sal* saliva volume. We assumed that a fraction *fsal_env* of the daily saliva secreted by an animal was deposited into the home-pen environment and a fraction *fsal_env_floor* of that landed on the floor. The total daily volume of saliva deposited into the home-pen floor by the clinical high infectious cattle was I_3_ × *sal* × *fsal*_*env* × *fsal*_*env*_*floor*, of urine it was I_3_ × *uri*, and of feces it was I_3_ × *fec*. The virus quantity shed by a highly infectious animal with clinical FMD per unit volume of saliva was *salv*, per unit volume of urine it was *uriv*, and per unit volume of feces it was *fecv*. The deposited secretions and excretions from the I_3_ were evenly distributed across the home-pen floor top in *j*. The viral decay in the resulting mixed floor materials occurred at a daily exponential rate *vir_dec_env*. The width of a home-pen was *w_pen*, the length was *l_pen*, and the contaminated floor top depth was *d_pen*. The remaining viral load in the contaminated floor-top materials in the home-pen *j* as

$$\begin{array}{l} FMDv\text{\_}floor_{j}\\ ={\left(\frac{{\text{I}}_{{\text{3}}_{j}}\times sal\times fsal\text{\_}env\;\times fsal\text{\_}env\text{\_}floor\times salv+{\text{I}}_{{\text{3}}_{j}}\times uri\times uriv+{\text{I}}_{{\text{3}}_{j}}\times fec\times fecv}{w\text{\_}pen\times l\text{\_}pen\times d\text{\_}pen}\right)}^{-vir\text{\_}dec\text{\_}env}. \end{array}$$

We assumed that an amount σ of the virus-containing floor materials from the originating (visited by pen-riders first) home-pen *j* was transported on the boots of the pen-riders or hooves of the horses during each pen-rider round to the next—receiving—home-pen *i* in the same row. The pen-rider rounds through the home-pen row occurred twice per day. In the receiving home-pen *i*, we assumed that the maximum number of cattle that could be infected due to consumption of the transported contaminated materials was $\frac{FMDv\text{\_}floor_{j}\times \sigma }{ID_{50}\text{ per oral}}$. The infections occurred on the same day when the materials were introduced to *i*. The daily number of cattle in *i* infected via this route was modeled as $Bin\left[\left(\frac{FMDv\text{\_}floor_{j}\times \sigma }{ID_{50}\text{ per oral}}\right),\;0.5\right]$ (Equations 1, 2). Definitions and values of the parameters are given in Table 1.

iv *Waterborne transmission*

We assumed that only contiguous home-pens which shared a drinking water-trough were at risk of waterborne FMDv transmission (see Supplementary Figures 1A–E for the feedlot layouts modeled). Potential waterborne transmission among home-pens that did not share a drinking water-trough was not modeled. Hospital-pens did not share drinking water-troughs with home-pens in feedlot layouts modeled. We assumed that a fraction (*fsal*) of the daily saliva secreted by an animal (*sal*) was deposited in the home-pen environment, of which a fraction *fsal_env_w* was deposited in the drinking water-trough. We assumed that all the saliva deposited into the home-pen environment was deposited in the water-trough or the floor, hence, *fsal*_*env*_*w* = 1−*fsal*_*env*_*floor*. We assumed that only saliva of the clinical high infectious cattle (I_3_) contributed to this transmission route. Daily volume of saliva produced by an animal, the fraction of the daily saliva volume deposited into the home-pen environment and what fraction of that deposited in the shared water-trough(s) by the I_3_ animals from the two home-pens that shared the water-trough, viral quantity shed per unit volume of saliva by an animal in the clinical high infectious FMD stage, volume of water in the shared water-trough, and viral decay in the water were reflected to model the remaining viral load in the water in the shared trough. A homogenous mixing of the deposited saliva with the water in the trough was assumed. The viral decay in the cattle drinking water occurred at a daily exponential rate *vir_dec_w*. The water volume in a shared trough was *vol_watert*. The home-pen *i* only shared a water-trough with one other home-pen *j* (here, either *j* or *h* could be on either side of *i*). If the home-pen *i* was at the end of the home-pen row in a row with odd number of home-pens, it did not share the water-trough with other home-pens and waterborne transmission was not modeled. The viral load per L of water in the water-trough shared by *i* and *j* was:

$$\begin{array}{l} FMDv\_watert_{i,j}={\left(\frac{({\text{I}}_{3_{i}}+{\text{I}}_{3_{j}})\times sal\times fsal\times fsal\_env\_w\times salv}{vol\_watert}\right)}^{-vir\_dec\_w} \end{array}$$

We assumed an animal consumed at least 1 L of water every time they visited the water-trough. On each day when *FMDv*_*watert*_*i, j*_ was ≥ID_50_ of FMDv for oral exposure, the number of cattle infected by FMD in the home-pen *i* via consumption of contaminated water from that shared trough was modeled as a *Bin*(S_*i*_, 0.5) (Equations 1, 2). Definitions and values of the parameters are given in Table 1.

v *Airborne transmission*

Airborne transmission was modeled using a kernel function that incorporated an exponential decay in the FMDv transmission probability with increasing Euclidian distance between home-pen centroids. Based on the feedlot layout detailed in Supplementary Figure 1, we estimated the Euclidean distance between centroids of a home-pen *i* and *k* (where *k* is any other home-pen than *i*) and scaled it by the shortest Euclidean distance between any two home-pen centroids in the feedlot. The scaled distance between two home-pen centroids was *d*_*i*__, *k*_. The airborne transmission probability to a home-pen *i* depended on the distances to and proportions of FMD clinical highly infectious cattle (I_3_) in the other home-pens. The proportion in a home-pen *k* was $\frac{{\text{I}}_{{\text{3}}_{\text{k}}}}{N_{k}}$. The probability of FMD infection of a susceptible animal in *i* via the airborne transmission was $p\text{\_}air_{i}=1-\left[\overset{n}{\underset{k=1}{\prod }}k\left(1-\frac{{\text{I}}_{{\text{3}}_{\text{k}}}}{N_{k}}\times e^{-\alpha \times d_{i,k}}\right)\right]$, and the daily number of cattle infected was *Bin*(S_*i*_, *p_air*_*i*_) (Equations 1, 2). Value of the parameter α reflected the power of the kernel function (Table 1).

### Outbreak Characteristics Analyzed

We defined the following characteristics of the projected FMD outbreaks, traced these outputs during the model simulations, and analyzed sensitivity of the outputs to the model structure and parameter values. The outbreak characteristics were:

- Outbreak peak day defined as the day with the highest number of clinical cattle (those in the I_3_ and C compartments) in the feedlot, counting from the day of introduction of FMD latent cattle into the index home-pen.
- Number of clinical cattle in the feedlot on the outbreak peak day.
- Day of outbreak detection in the feedlot, counting from the day of introduction of FMD latent cattle into the index home-pen. The detection was assumed to occur on the day when the proportion of clinical cattle in the index home-pen reached a detection threshold of 3, 5, or 10% (the lowest detection threshold of 3% was chosen based on data provided via personal communication by veterinarians with experience of FMD investigation on cattle farms during the FMD outbreaks in South America in the 2000s).
- Proportion of latent cattle in the feedlot on the day of outbreak detection.
- Cumulative number of infected home-pens (a home-pen was counted on the day when FMD latent cattle occurred in it for the first time) in the feedlot throughout the outbreak.
- Outbreak duration defined as the day when the last clinical infectious cattle became clinical non-infectious, counting from the day of introduction of FMD latent cattle into the index home-pen.

### Model Implementation, Verification, and Validation

The model was implemented in Vensim® PLE Plus Version 6.4a (Ventana Systems Inc., Harvard, MA, USA). The figures were made in R using the ggplot package and in Microsoft Office Power Point® 365 ProPlus (Microsoft, Redmond, WA, USA). The statistical analysis was done in STATA® 13 (StataCorp LP, College Station, TX, USA). Distances between home-pen centroids in each of the feedlot size and layout cases were estimated using Autodesk® Fusion 360 (Autodesk, Inc., San Rafael, CA, USA).

Model verification and validation were performed systematically during the model development and implementation process, i.e., after adding each new component, such as a virus transmission route or a new module, such as a section of the feedlot layout, and following recommended approaches (38, 39). Specifically, at each verification a dynamic approach described by Reeves et al. (39) was used to confirm the model behavior and outputs were logical when giving extreme parameter value inputs. A population balance check was done for the total number of cattle in the feedlot on each day simulated. We conducted a conceptual validation that the model met the intended purpose which was to project FMDv transmission and clinical manifestation dynamics within the feedlot by capturing the effects of the different processes reflected in the model, and a face validation which consisted of an assessment of the system modeled and model outputs by experts in epidemiological models (38, 39).

### Sensitivity Analyses of the Model Outputs to the Model Structure and Parameter Values

i *Sensitivity analysis of the model outputs to the index home-pen location within the feedlot and time spent by individual cattle in the hospital pen per visit*

Cattle with latent FMD were introduced into one home-pen; a proportion of cattle in the index home-pen were FMD-latent at the start of simulations on day 0. Three scenarios of the index home-pen location within the feedlot were modeled: S1—index home-pen was located at the edge of the feedlot and shared a drinking water-trough with one contiguous home-pen; S2—index home-pen was located at the edge of the feedlot and did not share a drinking water-trough with another home-pen; and S3—index home-pen was located centrally within the feedlot and shared a drinking water-trough with one contiguous home-pen. In the base scenario individual cattle pulled to the hospital-pen on one day returned to the home-pen on next day (the beta transmission parameter value for the FMD transmission via direct contact of cattle in the hospital-pen(s) per day was β_*hp*_). In a comparative scenario, cattle spent a half day in the hospital-pen, returning to the home-pen same day when pulled (the transmission parameter value was β_*hp*_/2). The model was simulated for each of the feedlot size and layout cases (detailed in Supplementary Figure 1) with each of the three scenarios of the index home-pen location within the feedlot, and each of the two scenarios of the time spent by individual cattle in the hospital-pen per pull due to a production disease or FMD. For each case and scenario, 2,000 Monte Carlo simulations were performed and the FMD outbreak characteristics (listed in section Outbreak Characteristics Analyzed) were traced during the simulations. After evaluating the model outputs and if there were no variations in the outputs, a base scenario of the index home-pen location and the time spent by individual cattle in the hospital-pen per visit was chosen based on closest representation to production systems. The base scenario was implemented in the remainder of the sensitivity analyses.

ii *Sensitivity analysis of the model outputs to the parameter values*

The model output sensitivity analysis to values of a set of target parameters was performed. Values from each of the target parameters were sampled for each of 2,000 Monte Carlo simulations of the model. The model was simulated for each specific feedlot size and layout case and the chosen base scenario of the index home-pen location and the time spent by individual cattle in the hospital-pen per visit. The sampled distributions of the target parameters are given in **Table 4**. For each of the remaining model parameters, a single value listed in Table 1 was used for each of the 2,000 simulations. The target parameters included the FMD latent, infectious, and subclinical periods in individual cattle and cattle infectivity as a change in the value of the beta transmission parameter within the home-pens, fence-line, and in the hospital-pen(s). The infection and disease temporal progression and infectivity could vary with the strain virulence (40). Thus, targeting these parameters in the sensitivity analysis allowed evaluating the model outputs for different FMDv strain virulence scenarios. The target parameter set also included the BRD morbidity in the first 30 days since the cattle placement in the feedlot. The morbidity increases the cattle pull rate to the hospital-pens, but it could vary depending on the feedlot production management and time of year. The target parameter set also included the initial proportion of FMD-latent cattle in the index home-pen, the fraction of daily saliva volume secreted by an animal that is deposited to the home-pen environment, the home-pen floor top depth contaminated by the animal secretions and excretions daily, the water intake per cattle visit to the drinking water-trough, the mortality rate for animals with BRD and other production diseases, the mortality rate for animals with clinical FMD, the urine volume produced by an animal, the saliva volume produced by an animal, the volume of feces produced by an animal, the virus quantity shed in urine by an animal in the FMD clinical high infectious status, the virus quantity shed in saliva by an animal in the FMD clinical high infectious status, the virus quantity shed in feces by an animal in the FMD clinical high infectious status, and the proportion of the cattle daily saliva volume deposited into the home-pen environment (Table 1).

Sensitivity to the values of the target parameters was analyzed for outbreak peak day with highest number of clinical cattle and outbreak duration in the feedlot. Using the outputs of the 2,000 model simulations for each of the feedlot size and layout cases, statistical significance of a pair-wise association between the value of each of the target parameters and each the outbreak peak day or outbreak duration was tested with the Spearman rank correlation coefficient. The pair-wise correlation was considered statistically significant if the *p-*value ≤ 0.05. Also using the simulation outputs, a multivariable linear regression model was built to identify a parameter group most associated with each of outbreak peak day and outbreak duration. A predictor variable was excluded from the model if *p-*value > 0.05 for its association with the outcome variable. The predictor variable selection was performed using the backward stepwise regression and the final model was chosen based on largest adjusted *R*^2^ value. The final multivariable linear regression model's adjusted *R*^2^ statistic was partitioned to obtain the fractional contributions of the target parameters to the projected outcome variance.

An additional parameter-value sensitivity analysis was performed for the power (α) of the function of an exponential decay in the probability of airborne FMDv transmission with increasing distance between home-pens (see the Kernel function definition in the section Model Formulation, subsection Airborne transmission). The model simulations were performed similarly to that described above for the target parameter set; additionally to sampling the value of each of the target parameters, the value of α (the sampled values are given in Table 3) was sampled for each of the 2,000 Monte Carlo simulations of the model. The model was simulated for each of the feedlot size and layout cases for each of the three-index home-pen location scenarios and assuming individual cattle spent 1 day in the hospital-pen pen visit. The outbreak duration distribution was summarized over the 2,000 simulated outbreaks with each value of α. The non-parametric Kruskal-Wallis test was used to test statistical significance of differences in the median outbreak duration with different values of α for a given scenario and for a given feedlot of size and layout case. If *p*-value ≤ 0.05 for the Kruskall-Wallis test, the Dunn's test with Bonferroni correction was conducted for the multiple comparisons.

iii *Relative impact of the FMDv transmission routes on the outbreak duration*

**Table 3:** Estimated percentage of latent cattle and home-pens with latent cattle on a U.S. beef cattle feedlot depending on the outbreak detection day since foot-and-mouth disease introduction.

**Feedlot^**a**^**	**Percentage (%) of latent cattle and home-pens with latent cattle in the feedlot on the day of FMD outbreak detection (10th, 50th, 90th percentiles of** ***n=*** **2,000 simulated outbreaks)**^****b****^					
		**Day 5**	**Day 6**	**Day 7**	**Day 8**	**Day 9**
FS1	Cattle	<1, 4, 7	1, 10, 14	2, 14, 18	6, 18, 24	13, 24, 25
	Home-pens	25, 25, 25	25, 25, 30	25, 30, 41	25, 35, 50	25, 50, 65
FM1	Cattle	<1, 1, 2	0, 3, 4	1, 5, 5	3, 6, 6	4, 6, 7
	Home-pens	7, 7, 8	7, 7, 8	8, 10, 13	8, 12, 18	8, 15, 25
FM2	Cattle	<1, 1, 2	0, 3, 4	1, 5, 5	3, 6, 6	4, 6, 7
	Home-pens	7, 7, 8	7, 7, 8	8, 10, 13	8, 10, 15	8, 13, 18
FL1	Cattle	<1, 1, 1	0, 2, 2	1, 2, 3	1, 3, 3	2, 3, 4
	Home-pens	3, 3, 4	3, 3, 4	4, 4, 7	4, 5, 8	4, 7, 11
FL2	Cattle	<1, 1, 1	0, 2, 2	1, 2, 3	1, 3, 3	2, 3, 4
	Home-pens	3, 3, 3	3, 3, 4	4, 4, 7	4, 5, 8	4, 7, 9

a * Feedlot sizes and layouts modeled are detailed in Table 2 and Supplementary Figure 1. Briefly, FS1 is a 4,000 cattle feedlot with one hospital-pen; FM1 is a 12,000 cattle feedlot with one hospital-pen; FM2 is a 12,000 cattle feedlot with two hospital-pens; FL1 is a 24,000 feedlot with two hospital-pens; and FL2 is a 24,000 cattle feedlot with four hospital-pens (in all the layouts n = 200 cattle per home-pen)*.

b *We show results of latent cattle and latent home-pens on days 5–9 (only) of outbreak detection on each feedlot size and layout modeled because those were the most common days of outbreak detection for the three detection thresholds modeled (3, 5, and 10% clinical cattle in the index home-pen)*.

The model structure sensitivity analysis was focused on the relative impact of the five routes of FMDv transmission between home-pens on the outbreak duration. The five routes were the direct animal contact in the hospital-pen(s), fence-line direct contact, via shared drinking water-troughs, via environment by pen-riders, and airborne. The model was simulated for each of the feedlot size and layout cases for each of the three-index home-pen location scenarios and assuming individual cattle spent 1 day in the hospital-pen pen visit. The value of each of the target parameters (Table 4) was sampled for each of the 2,000 Monte Carlo simulations of the model, while setting to zero the parameter values related to one of the transmission routes. The outbreak duration distribution was summarized over the 2,000 simulated outbreaks for the full model and each of the reduced models with one of the routes of transmission excluded, for each feedlot size-layout case and index home-pen location scenario. The non-parametric Kruskal-Wallis test was used to test statistical significance of differences in the median outbreak duration between the full and reduced models and for a given scenario for each of feedlot and layout cases. If *p*-value ≤ 0.05 for the Kruskall-Wallis test, the Dunn's test with Bonferroni correction was conducted for the multiple comparisons.

**Table 4:** Target parameters investigated for associations with the projected outbreak's peak day with highest number of clinical cattle since foot-and-mouth disease introduction and the total outbreak duration on a U.S. beef cattle feedlot.

Target parameter^*^	**Parameter value distribution**	**Strength of the correlation (Spearman correlation coefficient value) between the model parameter value and outcome variable value for the feedlot of that size and layout**									
		**Peak day of the outbreak**^****a****^					**Duration of the outbreak**				
		**FS1^**b**^**	**FM1**	**FM2**	**FL1**	**FL2**	**FS1**	**FM1**	**FM2**	**FL1**	**FL2**
Beta transmission parameter in home-pens (*β_*wp*_*)	Triangular (0.02, 0.026, 0.031)	−0.14^*^	−0.21^*^	−0.09^*^	−0.09^*^	−0.10^*^	−0.05^*^	−0.08^*^	−0.14^*^	−0.09^*^	−0.08^*^
Bovine respiratory disease morbidity during the first 30 days of cattle placement in the feedlot (π)	Vector (0.05, 0.30, 0.05)	−0.01	−0.05	0.03	−0.10^*^	−0.17^*^	−0.05^*^	−0.13^*^	−0.15^*^	−0.07^*^	−0.05^*^
Depth of the home-pen floor top contaminated by fresh animal excreta (*d_pen*) (m)	Vector (2, 5, 3)	−0.06	−0.05	−0.01	−0.04	−0.03	−0.05	−0.05	−0.05	−0.06	−0.06
Initial proportion of latent cattle in the index home-pen (*lat_initial*)	Vector (0.005, 0.105, 0.020)	−0.42^*^	−0.29^*^	−0.09^*^	−0.15^*^	−0.17^*^	−0.11^*^	−0.09^*^	−0.08^*^	−0.09^*^	−0.09^*^
Fraction of saliva daily produced by the animal that is excreted into the home-pen environment (σ)	Vector (0.1, 0.5, 0.1)	0	−0.05	0.03	−0.05	−0.03	0.04^*^	0.03^*^	−0.01^*^	−0.01^*^	0.01^*^
Duration of FMD latent period (*lat*) (days)	Weibull (α = 1.782, β = 3.974)	0.67^*^	0.62^*^	0.25^*^	0.48^*^	0.64^*^	0.75^*^	0.77^*^	0.77^*^	0.82^*^	0.83^*^
Duration of FMD infectious period (*inf*) (days)	Gamma (α = 3.969, β = 1.107)	0.02	−0.11^*^	−0.02	−0.14^*^	−0.12^*^	0.48^*^	0.42^*^	0.23^*^	0.35^*^	0.29^*^
Duration of FMD subclinical period (*sub*) (days)	Gamma (α = 1.222, β = 1.672)	0.19^*^	0.25^*^	0.07^*^	0.18^*^	0.22^*^	−0.21^*^	−0.17^*^	−0.03^*^	−0.09^*^	−0.06^*^
Water intake by the animal per visit to the water-trough in the home-pen (*wat_int*) (l)	Vector (1, 5, 4)	−0.02	−0.08	0.01	−0.09	−0.10	0.01	−0.01	−0.05	−0.06	−0.06

a Bold coefficients with ^*^ indicate p < 0.05 for the correlation coefficient between the parameter value and outcome variable value.

b *Feedlot sizes and layouts modeled are detailed in Table 2 and Supplementary Figure 1. Briefly, FS1 is a 4,000 cattle feedlot with one hospital-pen; FM1 is a 12,000 cattle feedlot with one hospital-pen; FM2 is a 12,000 cattle feedlot with two hospital-pens; FL1 is a 24,000 feedlot with two hospital-pens; and FL2 is a 24,000 cattle feedlot with four hospital-pens (in all the layouts n = 200 cattle per home-pen)*.

* *Results of the following target parameters were not included in the table above because were found to be not influential to model outputs: mortality rate for animals with BRD and other production diseases (endemic infectious diseases and noninfectious diseases) (day^−1^) (μ), Mortality rate for animals with clinical FMD (day^−1^) (ψ), urine volume produced by an animal (L/day) (uri), saliva volume produced by an animal (L/day) (sal), volume of feces produced by an animal (kg/day) (fec), virus quantity shed in urine [plaque forming units (PFU)/mL] by an animal in the FMD clinical high infectious status (uriv), virus quantity shed in saliva (PFU/mL) by an animal in the FMD clinical high infectious status (salv), virus quantity shed in feces (PFU/mL) by an animal in the FMD clinical high infectious status (fecv), and the proportion of the cattle daily saliva volume deposited into the home-pen environment (dmnl) (fsal_env). Their distributions can be found in Table 1*.

## Results

### Characteristics of Projected FMD Outbreaks in Feedlots of Different Sizes and Layouts

There was no significant variation in the outbreak characteristics among the three scenarios of the index home-pen location within the feedlot, in any of the feedlot size and layout cases modeled (see Supplementary Figure 1). There was also no significant variation in the outbreak characteristics when individual cattle were assumed to spend a full day vs. a half of day in the hospital-pen per visit, in any of the feedlot size and layout cases and index home-pen location scenarios. In the light of this, we present results of the FMD latent cattle introduced in an index home-pen that was located centrally within the feedlot, shared a drinking water-trough with one contiguous home-pen (S3 scenario) and cattle spending a full day in the hospital-pen per visit. For each feedlot size and layout scenario, 2,000 Monte Carlo simulations of the model were performed with sampling of the target parameters from the distributions specified in Table 1. In short, FS1 was a 4,000 cattle (20 home-pens) feedlot with one hospital-pen; FM1 was a 12,000 cattle (60 home-pens) feedlot with one hospital-pen; FM2 was a 12,000 cattle feedlot with two hospital-pens; FL1 was a 24,000 cattle (120 home-pens) feedlot with two hospital-pens; and FL2 was a 24,000 cattle feedlot with four hospital-pens.

**Figure 3:**
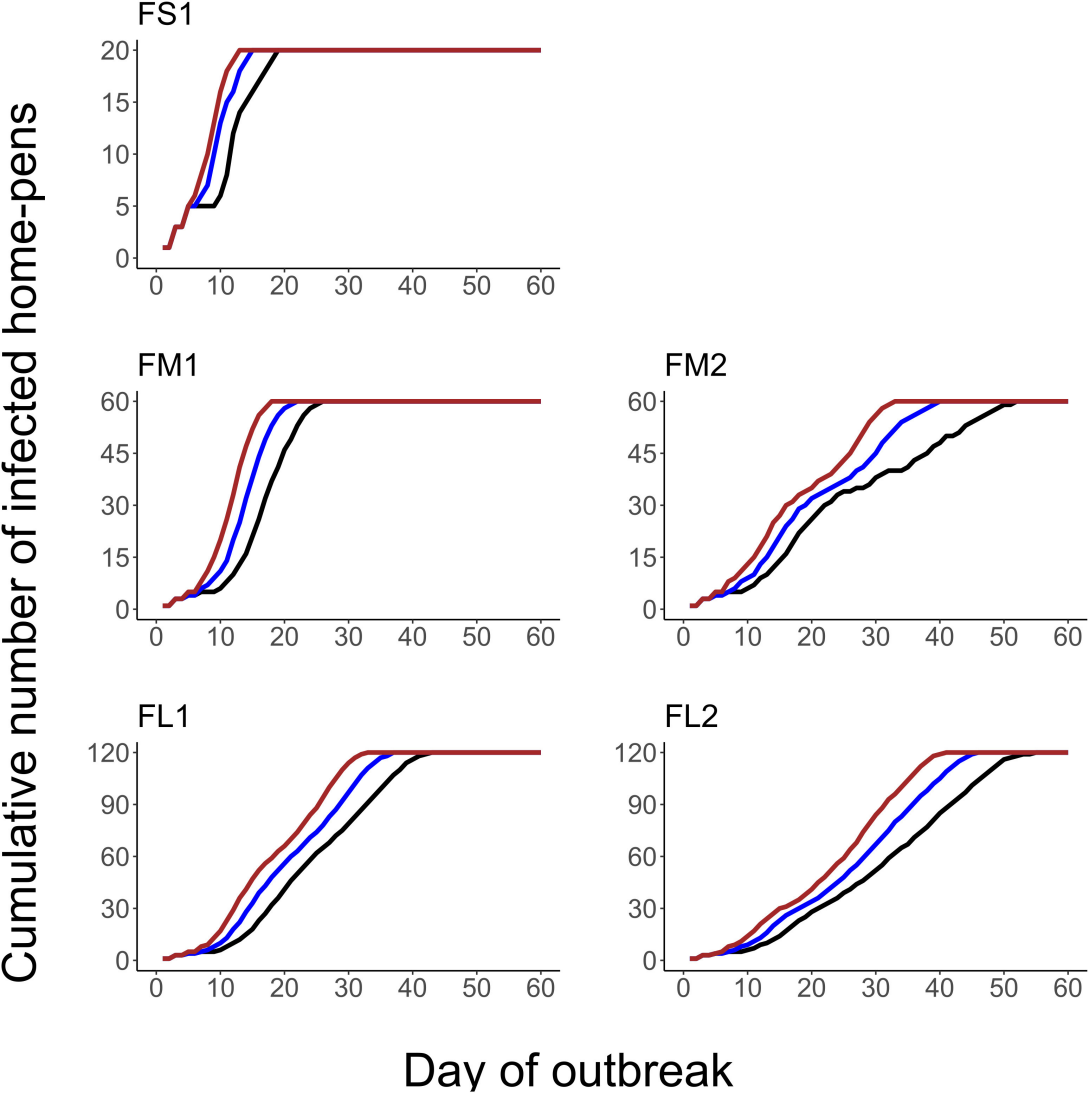
The cumulative number of the home-pens infected with foot-and-mouth disease during a projected outbreak on a U.S. beef cattle feedlot. The lines represent the percentiles (brown lines the 90th percentile, blue lines the 50th percentile, and black lines the 10th percentile) for *n* = 2,000 simulated outbreaks in the feedlot of that size and layout sampling the values of the target parameters. Feedlot size and layout cases modeled: FS1 is a 4,000 cattle feedlot with one hospital-pen; FM1 is a 12,000 cattle feedlot with one hospital-pen; FM2 is a 12,000 cattle feedlot with two hospital-pens; FL1 is a 24,000 feedlot with two hospital-pens; and FL2 is a 24,000 cattle feedlot with four hospital-pens (in all the layouts *n* = 200 cattle per home-pen).

**Figure 4:**
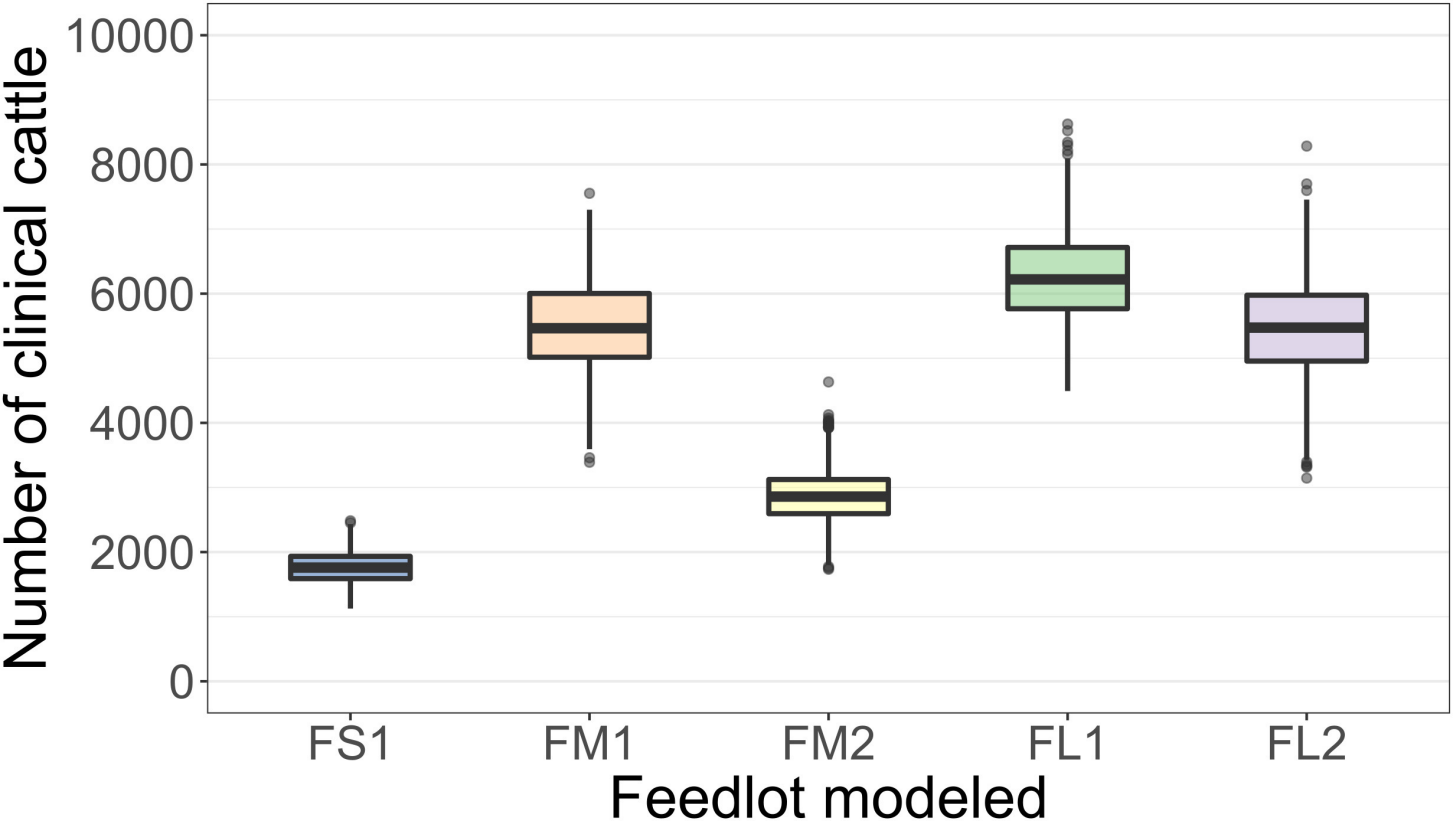
Boxplot of the projected number of cattle with clinical FMD on the outbreak peak day for each of the feedlot size and layout cases modeled. The outbreak peak day was defined as the day with the highest number of clinical cattle (infectious and non-infectious) since the FMD introduction in each of n = 2,000 simulated outbreaks in the feedlot of that size and layout sampling the values of the target parameters. Feedlot size and layout cases modeled: FS1 is a 4,000 cattle feedlot with one hospital-pen; FM1 is a 12,000 cattle feedlot with one hospital-pen; FM2 is a 12,000 cattle feedlot with two hospital-pens; FL1 is a 24,000 feedlot with two hospital-pens; and FL2 is a 24,000 cattle feedlot with four hospital-pens (in all the layouts n = 200 cattle per home-pen).

The projected outbreak duration ranged from 49 days in the smallest FS1 to 82 days in the largest FL2 feedlot. The outbreak peak day ranged from 23 in FS1 to 49 days in FL2. The outbreak proceeded slower and lasted longer in a feedlot of a given size if more hospital-pens were operated. The median outbreak duration was 16 days longer in a medium-size feedlot FM2 where 2 hospital-pens were operated compared to FM1 where 1 hospital-pen was operated (Table 2). In a large-size feedlot, the median outbreak duration was 9 days longer if two hospital-pens per section of home-pens were operated (four hospital-pens total, FL2), compared to one hospital-pen per section (two hospital-pens total, FL1) (Table 2). All home-pens were infected by day 15 following introduction of FMD latent cattle onto the feedlot in FS1, on day 22 in FM1 vs. day 40 in FM2, and on day 37 in FL1 vs. day 46 in FL2 case (Figure 3). The number of clinical cattle on the outbreak peak day decreased with a larger number of hospital-pens (Figure 4). The median number of clinical cattle on the outbreak peak day was 1,760 (44%) in FS1, 5,520 (46%) in FM1 vs. 2,880 (24%) in FM2, and 6,240 (26%) in FL1 vs. 5,520 (23%) in FL2. Thus, a higher number of hospital-pens had a larger impact on the FMD outbreak dynamics—slowing the outbreak and decreasing the percentage of clinical cattle on the peak day—in a medium-size (12,000 cattle) than in a large-size (24,000 cattle) feedlot, for the layouts modeled.

### FMD Outbreak Detection

The outbreak detection was assumed to occur on the day when the proportion of cattle with clinical FMD in the index home-pen reached 3, 5, or 10%. The detection timeline was therefore similar for all the feedlot size and layout cases. The results presented are for the base scenario of FMD latent cattle introduced in an index home-pen that was located centrally within the feedlot and shared a drinking water-trough with one contiguous home-pen, and when the pulled cattle spent a full day in the hospital-pen per visit. The results are summarized over the 2,000 model simulations for each feedlot size-layout case. The day of detection ranged from 4 to 12 days since introduction of FMD latent cattle in the index home-pen for 3 and 5% detection thresholds, and from 6 to 13 days for the 10% threshold (Table 2). The median day of detection was 6 for 3 and 5% detection thresholds, while it was 7 for the 10% threshold. Overall, the longer it took to detect the outbreak, the larger was the proportion of latent cattle in the feedlot at detection; however, the relative magnitude of this impact declined with the feedlot size. In ~50% of the simulations, the outbreak was detected on day 5–9 with any of the three detection thresholds modeled. The median proportion of latent cattle in the smallest FS1 feedlot increased from 4% at detection on day 5–24% on day 9, with 25 and 50% of home-pens infected, respectively. In both FM1 and FM2, the median proportion of latent cattle increased from 1% at detection on day 5 with 7% of home-pens infected to 6% on day 9 with 15% (FM1) and 13% (FM2) of home-pens infected. In both FL1 and FL2, the median proportion of latent cattle increased from 1% on day 5 with 3% of home-pens infected to only 3% on day 9 with 7% of home-pens infected (Table 3).

### Sensitivity of the Projected Outbreak Characteristics to the Model Parameter Values

**Figure 5:**
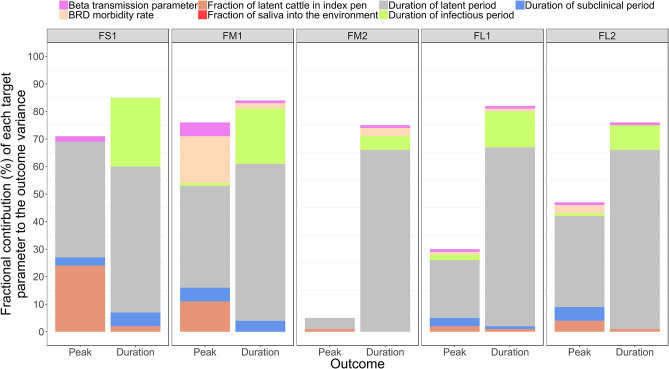
The fractional contributions of select target parameters to the variance in each the outbreak peak day with highest number of clinical cattle and the total outbreak duration in the feedlot since the foot-and-mouth disease introduction, estimated based on *n* = 2,000 simulated outbreaks in each of the feedlot size and layout cases modeled. Multivariable linear regression models were developed for each of the outcome variables of the projected outbreak peak day and outbreak duration and the target parameters as the predictor variables. For each outcome, the final regression model adjusted *R*^2^ statistic was partitioned to obtain the fractional contributions of the target parameters to the projected outcome variance. Outcomes: Peak—outbreak peak day, Duration—duration of the outbreak. Target parameters: beta transmission parameter for FMD virus transmission via direct cattle contact [Beta transmission parameter]; morbidity rate of bovine respiratory disease (BRD) during the first 30 days since cattle placement in the feedlot [BRD morbidity rate]; initial proportion of FMD latent cattle in the index home-pen [Proportion of latent cattle in index pen]; fraction of the daily saliva volume produced by an animal that is deposited into the home-pen environment [Fraction of saliva into environment]; and the durations of the FMD latent period [Duration of latent period], infectious period [Duration of infectious period], and subclinical period [Duration of subclinical period] in individual cattle. Feedlot size and layout cases modeled: FS1 is a 4,000 cattle feedlot with one hospital-pen; FM1 is a 12,000 cattle feedlot with one hospital-pen; FM2 is a 12,000 cattle feedlot with two hospital-pens; FL1 is a 24,000 feedlot with two hospital-pens; and FL2 is a 24,000 cattle feedlot with four hospital-pens (in all the layouts *n* = 200 cattle per home-pen).

The sensitivity analysis was performed for the base scenario detailed above. Of the target parameters, the durations of the FMD infection stages in individual cattle were most influential on the outbreak duration and outbreak peak day in the feedlot (Table 4 and Figure 5). Using the simulation outputs, a multivariable linear regression model was built for each the outbreak duration and peak day of outbreak variables with the target parameters as the predictor variables (Table 4). The duration of the FMD latent period was the most influential parameter. The fractional contribution of the latent period duration to the variance in the outbreak duration ranged from 53% in FS1 to 66% in FM1, and to the variance of the outbreak peak day it ranged from 4% in FM2 to 42% in FS1 (Figure 5). The duration of the FMD infectious period was the second most influential parameter. Its fractional contribution to the variance in the outbreak duration ranged from 20% in FM1 to 25% in FS1. The infectious period contribution to the outbreak duration variance decreased with a larger feedlot size and for a feedlot of a given size it decreased if more hospital-pens were operated. This contribution was 25% for FS1, 20% for FM1 vs. 5% for FM2, and 13% for FL1 vs. 9% for FL2 (Figure 5). The subclinical period was less influential compared to the latent and infectious periods, with a fractional contribution of 5% or less to the variances of both the outcomes in all feedlots modeled.

A larger value of the beta transmission parameter (β_*wp*_) reflected a higher cattle infectivity for FMDv transmission via direct contact in the home-pens, fence-line, and in hospital-pen(s). A larger value of this parameter was negatively correlated with each the outbreak duration and outbreak peak day (Table 4). This appears straightforward that a higher virus transmission rate via direct animal contact could lead to a faster outbreak progression. However, the relative contribution of β_*wp*_ to the total variance in either the outbreak duration or outbreak peak was ≤ 5%, being low compared to that of the durations of the FMD infection stages in individual animals (Figure 5). The initial proportion of FMD-latent cattle in the index home-pen had smaller fractional contributions to the variances in the outbreak duration and outbreak peak day compared to the durations of the FMD stages and the beta transmission parameter (Figure 5). The contribution of the initial FMD-latent proportion in the index home-pen to the outbreak duration decreased with a larger feedlot size and in a medium-size feedlot was lower if more hospital-pens were operated. This contribution was 24% for FS1 and 11% for FM1, but it was <4% for FM2 and both FL1 and FL2 (Figure 5). The morbidity rate of BRD during the first 30 days since cattle placement in the feedlot was weakly correlated with both the outcome variables in each of the feedlot size and layout cases (Table 4). The fractional contribution of the BRD morbidity to the variance in the outbreak duration ranged from 1% in FL1 to at most 17% in FM1 (Figure 5).

The target parameter set for the sensitivity analysis (Table 4) included the parameters values that were initially assigned based on our judgment in the absence of data (Table 1). These were the fraction of daily saliva secreted by an animal that is deposited to the home-pen environment; the home-pen floor top depth daily contaminated by the animal secretions and excretions; and the water intake per cattle visit to the drinking water-trough. The values of each of these parameters had low correlations with the outbreak duration and outbreak peak day (Table 4), and low fractional contributions to the variances in these outcomes (Figure 5) across the feedlot size and layout cases. The remainder of the investigated target parameters (Table 4) were not influential for the two outcomes (results not shown) and are not discussed further. These were: the mortality rate for animals with BRD and other production diseases; the mortality rate for animals with clinical FMD; volumes of urine, saliva, and feces produced daily by an animal; proportion of the cattle daily saliva volume deposited into the home-pen environment; and the virus quantities shed in urine, saliva, and feces by an animal in the FMD clinical high infectious stage.

### Relative Impact of Individual Routes of FMDv Transmission Between Home-Pens on the Outbreak Duration

**Figure 6:**
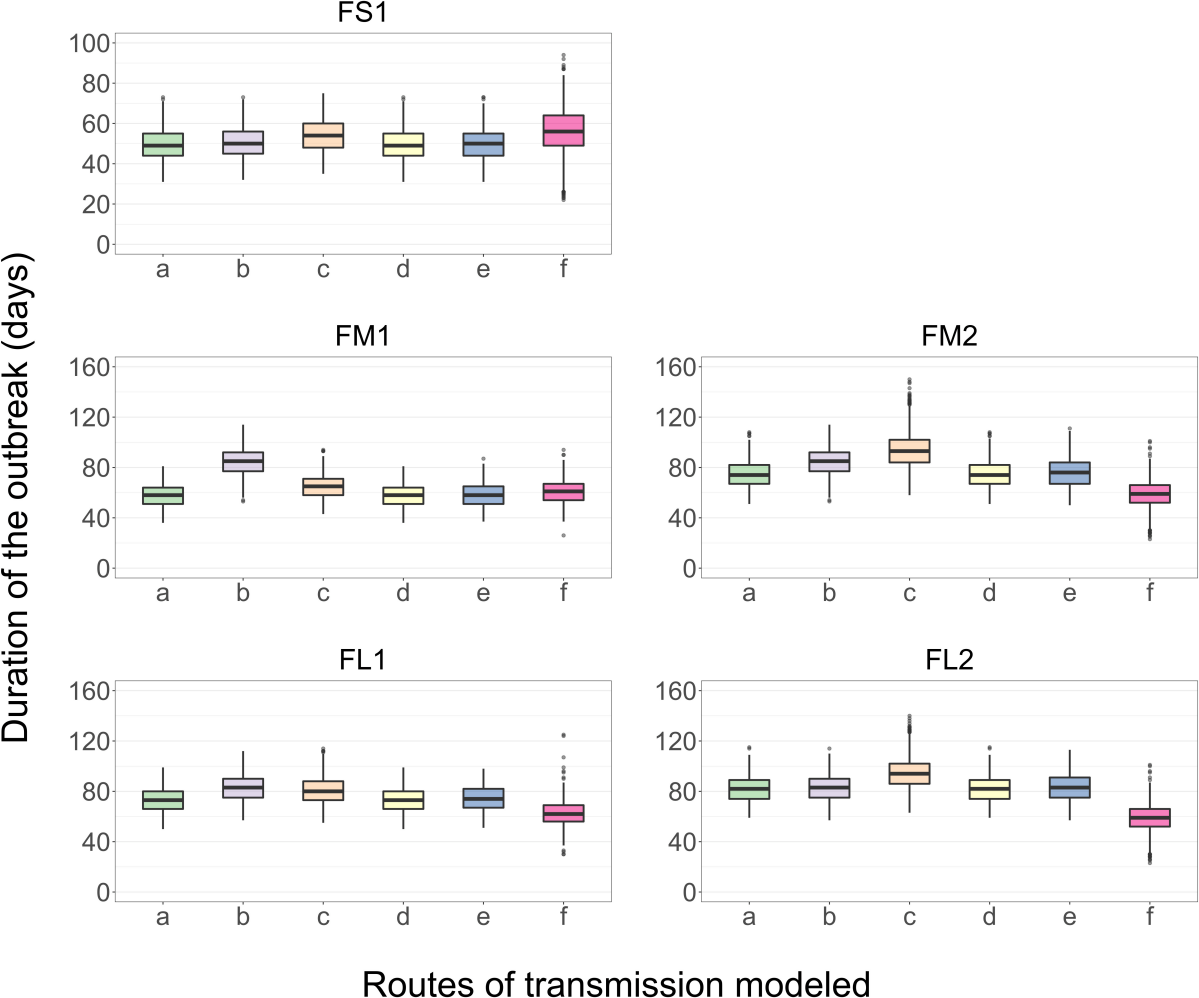
Boxplot of the projected duration of a foot-and-mouth disease outbreak on a U.S. beef cattle feedlot for *n* = 2,000 simulated outbreaks in the feedlot of that size and layout sampling the values of the target parameters, when the full model incorporating all the routes of FMD virus transmission among the home-pens or a model with one of the transmission routes excluded was simulated. a—all the routes of FMD virus transmission among home-pens incorporated, b—transmission via direct contact of cattle in the hospital-pens excluded; c—fence-line transmission between cattle in neighboring home-pens excluded; d—transmission of virus contaminated material between home-pens by the pen-riders excluded; e—transmission via contaminated water-troughs excluded; and f—airborne transmission excluded. Feedlot size and layout cases modeled: FS1 is a 4,000 cattle feedlot with one hospital-pen; FM1 is a 12,000 cattle feedlot with one hospital-pen; FM2 is a 12,000 cattle feedlot with two hospital-pens; FL1 is a 24,000 feedlot with two hospital-pens; and FL2 is a 24,000 cattle feedlot with four hospital-pens (in all the layouts *n* = 200 cattle per home-pen).

The results presented are for the base scenario detailed above. For each feedlot size-layout case, 2,000 model simulations were performed with sampling the values of the target parameters (Table 4), and also setting to zero the parameter values related to one of the between-pen FMDv transmission routes. Exclusion of the transmission via environment by pen-riders or the transmission via contaminated drinking water in the shared water-troughs did not result in a substantially different median outbreak duration or outbreak peak day (each *p* > 0.05 for the *post-hoc* multiple comparisons test) compared to that in the full models across the feedlot size and layout cases (Figure 6). Exclusion of the FMDv transmission via direct contact of cattle from different home-pens in the hospital-pen(s) resulted in a significantly longer median outbreak duration (*p* < 0.001 for the *post-hoc* multiple comparisons test) in FM1, FM2, and FL1 compared to the full models. The median outbreak duration in FM1 was 27 days longer, in FM2 it was 11 days longer, and in FL1 it was 10 days longer if the β_hp_ was set to 0 (Figure 6). Exclusion of the FMDv transmission via fence-line direct contact of cattle from contiguous home-pens resulted in a significantly longer median outbreak duration (*p* < 0.001 for the *post-hoc* multiple comparisons test) in all the feedlot size and layout cases, with largest differences in FM2, FL1, and FL2. Specifically, the median outbreak duration in FM2 was 19 days longer, in FL1 it was 7 days longer, and in FL2 it was 12 days longer (Figure 6). Exclusion of the airborne FMDv transmission resulted in a significantly shorter or longer median outbreak duration (*p* < 0.001 for the *post-hoc* multiple comparisons test), depending on the feedlot size and layout. The median outbreak duration in FS1 was 6 days longer, in FM1 it was 3 days shorter, but in FM2 it was 15 days shorter, in FL1 it was 11 days shorter, and in FL2 it was 23 days shorter (Figure 6).

### Impact of the Power (α) of the Function of an Exponential Decay in the Probability of Airborne FMDv Transmission With Increasing Euclidean Distance Between Home-Pen Centroids on the Outbreak Duration

**Figure 7:**
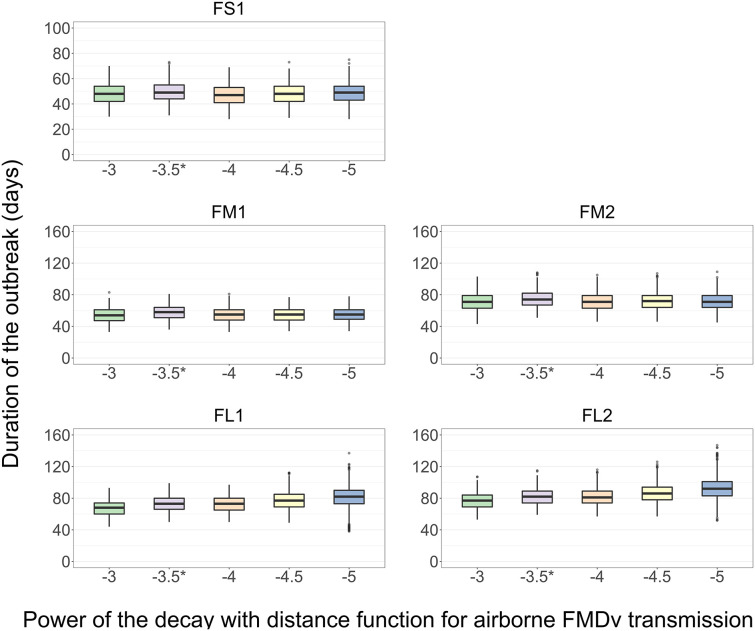
Boxplot of the projected duration of a foot-and-mouth disease outbreak on a U.S. beef cattle feedlot for *n* = 2,000 simulated outbreaks in the feedlot of that size and layout sampling the values of the target parameters, depending on the power (α) of the function of an exponential decay in the probability of airborne FMD virus transmission with increasing distance between home-pens. *Baseline value used to simulate the models for the other analyses. Feedlot size and layout cases modeled: FS1 is a 4,000 cattle feedlot with one hospital-pen; FM1 is a 12,000 cattle feedlot with one hospital-pen; FM2 is a 12,000 cattle feedlot with two hospital-pens; FL1 is a 24,000 feedlot with two hospital-pens; and FL2 is a 24,000 cattle feedlot with four hospital-pens (in all the layouts *n* = 200 cattle per home-pen).

The results presented are for the base scenario detailed above. For each feedlot size-layout case, 2,000 model simulations were performed with sampling the values of the target parameters (Table 4). Additionally, for each simulation a different power [α, modified from Boender et al. (24)] was specified for the Kernel function of an exponential decay in the probability of airborne FMDv transmission with increasing distance between home-pen centroids. The values of α modeled were: −3, −3.5 (baseline), −4, −4.5, and −5; a higher value of α represents a higher intensity of the airborne transmission. There was no significant difference in the median outbreak duration (*p* > 0.05 for the *post-hoc* multiple comparisons test) in FS1, FM1, or FM2 with a change in the value of α (Figure 7). In each FL1 and FL2, the outbreak duration was shorter with a higher value of α. In FL1, the median outbreak duration was 68 days with the highest α of −3 and 82 days with the lowest α of −5 (Figure 7). Similarly, in FL2 the median outbreak duration was 77 days with α of −3 and 92 with α of −5 (Figure 7).

## Discussion

The initial parameter values were assigned based on available data (Table 1). The available data are often for the serotype O that is the most prevalent serotype world-wide (2, 41) and responsible for recent epidemics in non-endemic countries with large livestock populations, such as the UK, France, Netherlands, South Korea, and Japan (42–47). However, the durations of the infection and disease stages in cattle can vary among FMDv strains, e.g., depending on the strain virulence (40). Other strain characteristics can also vary, e.g., transmissibility via direct animal contact (reflected by the β transmission parameter value) or airborne (reflected by the *a* parameter value in the airborne transmission Kernel function). We analyzed sensitivity of the projected outbreak duration and peak day to potential differences in the FMD strain characteristics associated with different disease period durations. A longer FMD latent period in individual cattle was associated with a later outbreak peak day and longer outbreak duration in all the feedlot size and layout cases modeled (Table 4). The transmission can be delayed since it takes longer for the animals to become infectious. A longer infectious period was also moderately correlated with outbreak duration and showed a weak negative correlation with days to peak infection in FM1, FS1, and FS2. A paper published after development of this model used experimental data and application of an Accelerated Failure Time model to estimate FMD disease periods and 95% confidence intervals but not fitted distributions (48). Their estimates are contained within the bounds of our model parameters for disease periods derived from Mardones et al. (32). Notably their point estimate of the latent period is shorter and of the infectious period is longer than our point estimates. In each case our distribution includes the estimates from Yadav et al. (48). Our sensitivity analysis suggests that both the latent period and the infectious period may be influential in outbreak dynamics. A shorter latent period may decrease the time to peak outbreak and increase the duration of the outbreak and a longer infectious period my increase the duration of the outbreak. Overall, these results suggest that characteristics of the FMDv strain will likely impact the transmission dynamics within the feedlot and outbreak characteristics. Given that the last documented FMD outbreak in the U.S. was in 1929 (4), an introduction of any FMDv strain would severely impact the U.S. livestock sector due to costs of the associated restrictions on international trade, animal depopulation or other control measures, and production losses (49).

The routes of direct and indirect FMDv transmission between home-pens in the feedlot were explicitly reflected in the model. The virus transmission from cattle with clinical and subclinical FMD via direct contact with susceptible cattle from other home-pens occurred in the hospital-pen(s) and fence-line for contiguous home-pens, along with the indirect waterborne, environmental, and airborne transmission. Of all the direct and indirect between-home-pen transmission routes, the direct transmission in the hospital-pen(s) had the largest impact on the outbreak duration in the median and large size feedlots that operated one hospital-pen per home-pen section (FM1 and FL1) (Figure 6). In the medium and large feedlots that operated two hospital-pens per section (FM2 and FL2), the fence-line direct transmission had the largest impact on the outbreak duration (Figure 6). Note that while the FMDv transmissibility via direct contact with infectious subclinical and clinical cattle and the effective contact rates were assumed to be equal in the home-pens and hospital-pens (β_*wp*_ = β_*hp*_), a simplified assumption was made that the fence-line contact rate was ¼ of the within home/hospital-pen rate (**β_*bp*_ = β_*wp*_ × 0.25**). The detailed role of the fence-line transmission can be explored in future models. We assumed equal FMDv transmissibility via direct contact from infectious subclinical and clinical cattle, because experimental studies show the virus shedding to the environment starts before the clinical signs (50–55). Such parameterization could lead to an overestimation of the within-herd FMD transmission rate as suggested by Kinsley et al. (31). To avoid the overestimation, the subclinical infectiousness and clinical infectiousness durations in our model were limited to the total infectious period reported by experimental and field studies (Table 1). The explicit specification of the subclinical and clinical infectious stages can be used in the future to investigate the contribution of animals in each of the stages to the transmission dynamics, if data on the FMDv shedding in excretions and secretions in subclinical and clinical animals become available. Moreover, the developed model structure with the explicit infection/infectiousness vs. clinical disease progression timelines (Figure 2) enables investigating the impact of the strain characteristics (e.g., the sensitivity analysis reported in Table 4 and Figure 5) as well as of specific vaccine formulations and vaccination strategies on the outbreak dynamics.

For the routes of indirect FMDv transmission—waterborne, environmental by pen-riders, and airborne—we only considered the contribution of cattle at the clinical high-infectious stage, because these are known to shed the virus in all the relevant excretions and secretions (55–57). The amount of virus shed by cattle with clinical FMD has been previously reviewed (35, 58–60). Data are extremely scarce on the shedding and other parameters relevant for FMDv indirect transmission in a feedlot; this is also relevant for the risk of transmission to other farms. We made a number of simplifying assumptions to model the indirect transmission in a feedlot. For the environmental transmission, we only considered the transmission of FMDv contaminated home-pen floor materials by pen-riders. We made a simplifying assumption that the FMDv containing animal secretions and excretions were evenly distributed across the home-pen floor, though this is unlikely. We assumed a floor material volume carried by a pen-rider between the home-pens, and the infectivity of the contaminated materials for cattle based on an infectious dose via per oral exposure (Table 1). To model FMDv transmission via drinking water in the water-troughs shared by contiguous home-pens, we made a simplifying assumption of an equal water volume consumed per visit to the trough by a healthy animal and an animal with FMD, and assumed the infectivity of the contaminated water for cattle based on an infectious dose via per oral exposure (Table 1). We did not model a specific drinking behavior, which is variable among cattle (61), season-dependent, and may change depending on the FMD stage. The drinking and feeding behavior changes during the FMD progression in cattle have not been sufficiently described in literature to enable inclusion in the model; future models could incorporate such data. Within limits of the current model structure and parameterization, the sensitivity analysis showed that neither the environmental FMDv transmission by pen-riders nor the transmission via contaminated drinking water substantially contributed to the projected outbreak duration (Figure 6). Other routes of indirect FMDv transmission, e.g., via contaminated fomites or personnel movement other than the pen-riding, may contribute to the transmission dynamics in feedlots but were not reflected in the model due to the lack of data for the parameterization.

The airborne FMDv transmission was influential on the outbreak duration (Figure 6). In the small FS1 feedlot, without the airborne transmission the projected outbreak duration varied significantly (Figure 6). This suggests the airborne transmission may contribute to a rapid and short outbreak in such feedlots with close spatial proximity of the home-pens. In the feedlots with multiple sections of home-pens (FM2, FL1, and FL2) in which 1–2 hospital-pens were operated for each home-pen sections, the airborne transmission was the only route of FMDv transmission responsible for the virus spread between the sections of home-pens. Without such transmission, only the index home-pen section was affected producing a shorter outbreak while the other home-pens sections remained uninfected. Hagerman et al. (62) showed that weather conditions are permissive of airborne FMDv spread in parts of the U.S. with significant beef cattle populations. However, no data is available on the expected intensity of the spread. To model a decreasing probability of airborne FMDv transmission with an increasing distance between home-pens in a feedlot, we adopted a Kernel function and its parameter values fitted by Boender et al. (24) to data from the UK 2001 FMD epidemic. This was an approximation since the parameter values were for the total probability of FMD spread via all routes among the cattle herds in the UK. To evaluate significance of this approximation, we investigated the impact of varying the key parameter of the function (the power α of the exponential decay in the airborne transmission probability with an increasing distance between home-pens) on the projected outbreak duration. The average outbreak duration was not significantly affected (Figure 7). However, in the large feedlots (FL1 and FL2) the outbreak duration was more variable when there was a lower probability of the airborne FMDv transmission via a given distance (a lower *a* value) (Figure 7). This suggests the airborne transmission can contribute to more predictable, shorter outbreaks even in larger feedlots. A simulation study by Donaldson and Alexandersen (63) showed a 100 infected cattle at a source would be enough for the virus to travel up to 1 km and infect a susceptible host which might suggest that within a medium to large beef feedlot, airborne transmission by itself can be responsible to the FMDv spread to the entire population. The airborne transmission might play a large role also for FMD spread between U.S. beef feedlots, because of the concentration of cattle farms within defined geographical areas, such as the Central United States where the majority of cattle is concentrated (62, 64). Environmental conditions however severely impact the airborne FMDv survival and transmission, and in turn depend on factors, such as seasonality and geographical location of the feedlot within the country (62).

The initial proportion of latent cattle in the index home-pen varied between a 0.5 and 10% and the BRD morbidity rate were not influential on the outbreak duration or peak day (Table 4 and Figure 5). We considered the cattle pulled to the hospital-pen(s) due to BRD as the main risk factor for contact of cattle from different home-pens during the first 30 days of the FMD outbreak. In our model, FMD was introduced with cattle arriving on the feedlot; the first 30 days post-arrival is on average the highest risk period to develop BRD in beef feedlots (65–68). Although, that risk period can be affected by several other factors (69–72) that were not further reflected in our model. Beyond designating at the start of the simulations some of the home-pens as just placed and the remainder as placed >30 days prior—to model the post-arrival BRD morbidity—we did not explicitly model the endemic disease incidence dependent on days on feed. Cattle in all the home-pens experienced an equal incidence of common production diseases other than BRD throughout the simulated outbreak. Realistically, cattle arrive on and leave the feedlot on a continuous basis, as a home-pen in/home-pen out.

**Figure 8:**
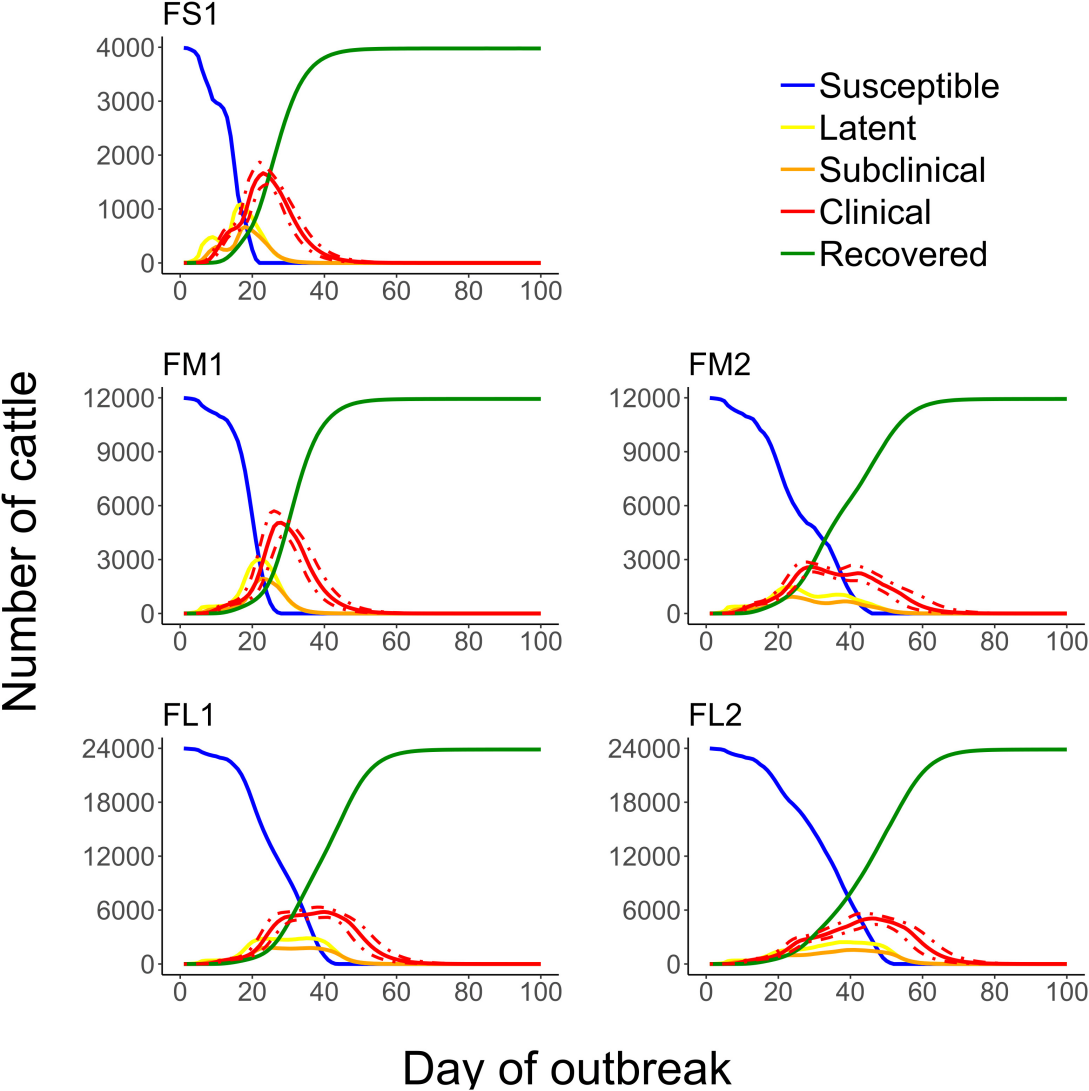
Numbers of cattle in each of the foot-and-mouth disease infection and disease stages during projected outbreaks on U.S. beef cattle feedlots. The solid lines represent the 50th percentiles for the cattle numbers in the infection stages and the red dotted lines represent the 25th and 75th percentiles for the number of cattle with clinical FMD (infectious and non-infectious clinical cattle) of *n* = 2,000 simulated outbreaks in the feedlot of that size and layout sampling the values of the target parameters. Feedlot size and layout cases modeled: FS1 is a 4,000 cattle feedlot with one hospital-pen; FM1 is a 12,000 cattle feedlot with one hospital-pen; FM2 is a 12,000 cattle feedlot with two hospital-pens; FL1 is a 24,000 feedlot with two hospital-pens; and FL2 is a 24,000 cattle feedlot with four hospital-pens (in all the layouts *n* = 200 cattle per home-pen).

The feedlot layout and number of hospital-pens operated impacted on the FMD outbreak characteristics. The projected FMD outbreak duration was shortest for feedlots with one hospital-pen serving one section of home-pens (Figure 8), because cattle from the whole feedlot mixed in a single hospital-pen. For medium or large feedlots (12,000 and 24,000 cattle, respectively), operating a lower number of hospital-pens resulted in a shorter outbreak (Figure 8). The outbreak peak day occurred earlier in feedlots with one hospital-pen and there was a large burden of the FMD clinical cattle earlier in the outbreak and on the outbreak peak day (FS1 and FM1) (Figures 4, 8). The epidemic curves were bi-modal in feedlots with more than one hospital-pens (FM2, FL1, and FL2); limiting differences in the number of cattle in the clinical stage during the outbreak. Overall, for a feedlot of a given size, the number of clinical cattle at the outbreak peak day(s) was lower with more hospital-pens operated (FM2 vs. FM1, FL2 vs. FL1) (Figure 4), which can be a result of the delayed outbreak progression due to the segregation of the hospital-pen catchment sub-populations of cattle. However, all the home-pens were infected during the outbreak in all the feedlots modeled, despite the differences in the cattle population size, number of home-pens sections per hospital-pen, or number of hospital-pens. These results suggest that a reduction of cattle contact within the feedlot by operating multiple hospital-pens, each with a defined catchment home-pen sub-population, might slow down the outbreak progression. This would provide time for implementing outbreak control strategies and reduce the FMD clinical cattle burden on individual days. On the other hand, operating a lower number of hospital-pens might lead to a faster and shorter outbreak. If no intervention strategies are implemented, a rapid outbreak progression might be the best scenario, with a lower risk of FMD transmission to other farms.

The outbreak was detected on day 4–12 since introduction of FMD latent cattle on the feedlot, if it was detected at 3% of clinical FMD cattle in the index home-pen (Table 2). The time to FMD detection in cattle herds was estimated to be 21 days during the UK 2001 epidemic (42) and 13 days during the 2010/2011 Korean epidemic (73). McLaws and Ribble (74) reviewed the time to detection for FMD outbreaks in livestock in non-endemic areas during 1992 to 2003; it varied from 7 to 24 days and reasons for a delayed detection included misdiagnosis of the disease, mild clinical signs (in small ruminants), delayed laboratory confirmation, and deliberate underreporting by the affected farmers. Prior modeling studies of FMD dynamics in livestock herds suggested the mean time to detection to be 10–11 days (17), 6–7 days (13), and 10–13.5 days (29) since FMD introduction. We modeled the day of detection based on identification of FMD clinical signs by the pen-riders during the routine observational surveillance. Pen-riders represent the first line of surveillance as they monitor cattle for clinical signs of endemic diseases within the feedlots, and are generally experienced in identifying diseased cattle (75). However, it is important to consider the differential diagnosis as there are cattle diseases with similar symptomatology as mentioned by Coetzer and Tustin (56); misdiagnosis can delay the time to detection in the field. The clinical disease severity also depends on the FMDv strain virulence (35, 56). To account for potential delays in the detection, we also considered the detection thresholds of 5 and 10% of FMD clinical cattle in the index home-pen. The outbreak detection was delayed by only 1–2 days for detection at 5 or 10% compared to 3% of FMD clinical cattle (Table 2). Nelson et al. (55) suggests the possibility to use qPCR to identify FMDv in cattle during the pre-clinical stage. The use of a surveillance test detecting pre-clinical FMD could potentially decrease the time to detection, however, no such pen-side (practical) test for cattle is currently available. The model simulations suggest that proportion of latent cattle in the feedlot can substantially increase from day 4 to 12 of the outbreak (Figure 8 and Table 3). This had a larger impact in small-size feedlots which in the worst-case scenario of detection on day 12 had up to 28% of the cattle already infected (data not shown). Carpenter et al. (29) modeled FMD transmission within a 1,000-cattle dairy farm; the results suggested 65–97% of the cattle would be infected by the day of detection at a 1 and 5% clinical FMD prevalence, respectively. However, the animal contact structure in dairy farms differs from that in beef feedlots. Studies modeling within-farm FMD dynamics have shown that early detection has a large impact on the scale of the outbreak and the success of intervention strategies (15, 16, 23).

We modeled feedlots as a closed system in which incoming and outgoing animals during the simulations were not considered. While U.S. feedlots generally have continuous turnover of cattle, once FMD was diagnosed quarantine would result in quarantine of the infected feedlot.

To our knowledge, this is the first model of transmission dynamics of FMD in beef feedlots. Kinsley et al. (31) modeled FMD transmission dynamics in swine farms. They estimated an earlier outbreak peak day—with highest number of clinical animals—on a swine farm compared to our estimate for a feedlot. This may be due that swine shed FMDv in larger quantities to the environment compared to cattle (35, 51, 56); this can contribute to the rapid infection transmission across the farm. The within-farm animal contact structure differs between swine farms and beef feedlots, and it can be expected that the FMDv transmissibility via different routes varies due to the different animal contact structure, virus shedding, and potentially virus survival in the farm environment. However, the estimated average time to FMD detection on a swine farm based on observation of the clinical signs was 3–12 days post-introduction (31), which is similar to day 4–12 in our model for beef feedlots (Table 2).

## Conclusions

This is the first model projecting FMD transmission, infection, and clinical manifestation dynamics on contemporary U.S. beef cattle feedlots. The model is consistent with data available to date but can be improved with better data on FMDv survival in within-feedlot environments (e.g., in cattle manure and drinking water); FMDv infectious dose depending on the exposure route for cattle that are healthy or experience common production diseases; clinical presentation of FMD in beef feedlot cattle depending on the strain virulence; potential for the virus airborne transmission in areas where the U.S. beef industry is concentrated; and sensitivity of the routine observational surveillance of large cattle populations to detect FMD introduction. Also, the modeling results highlight the importance of understanding the complex contact structure in the cattle meta-populations within feedlots for projecting possible dynamics of FMD and other infectious diseases. The lack of such understanding limits the realism and granularity of current models of within-farm dynamics of foreign animal diseases if (re)introduced to the U.S. The developed model will be used to project and compare impacts of FMD control strategies, such as cattle depopulation, within-feedlot movement restrictions, and vaccination on the outbreak progression. Finally, we emphasize that although mathematical models are powerful tools to understand complex systems, they are simplified representations of real life.

## Data Availability Statement

The datasets generated for this study are available on request to the corresponding author.

## Author's Note

This work was adapted from a doctoral dissertation chapter available online at: https://krex.k-state.edu/dspace/handle/2097/40307.

## Author Contributions

VV conceived the study. MS and VV designed the study. AC implemented the models and performed the sensitivity analyses. All authors contributed to the development, implementation, analysis of the models and the output interpretation, wrote the manuscript, read, and approved the final version for publication.

## Conflict of Interest

The authors declare that the research was conducted in the absence of any commercial or financial relationships that could be construed as a potential conflict of interest.
