# Supplementary material for: A Meta-Population Model of Potential Foot-and-Mouth Disease Transmission, Clinical Manifestation, and Detection Within U.S. Beef Feedlots
Source: Front Vet Sci. 2020 Sep 23;7:527558. doi: 10.3389/fvets.2020.527558 (PMC7543087; doi:10.3389/fvets.2020.527558)
Supplement: Supplementary file 1 [file Data_Sheet_1.docx]

**SUPPLEMENTARY MATERIALS**

**Supplementary Figure 1: Feedlot layouts modeled.**

**
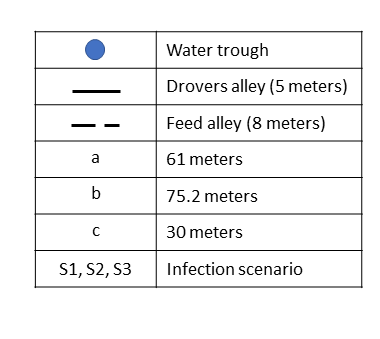
**

| **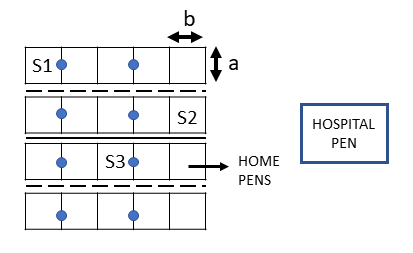** |  |
| --- | --- |

**Figure S 1A** – FS1 small-size feedlot with one hospital-pen.

| **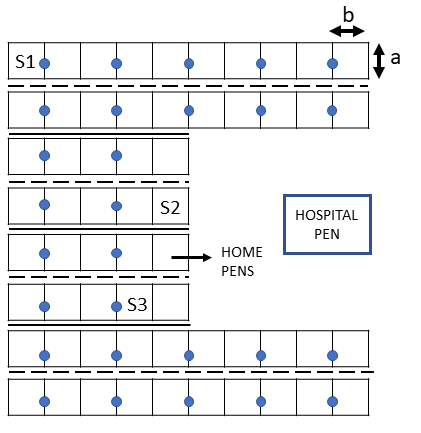** |
| --- |

**Figure S 1B –** FM1 medium-size feedlot with one hospital-pen.

| **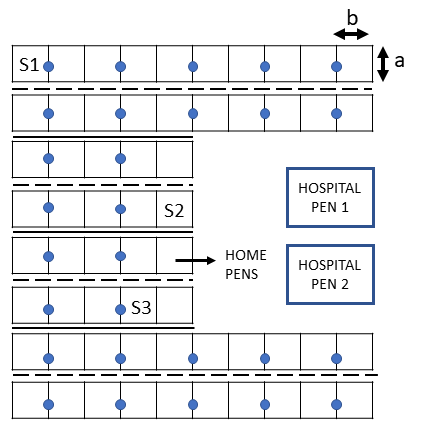** |
| --- |

**Figure S 1C** – FM2 medium-size feedlot with two hospital-pens.

| **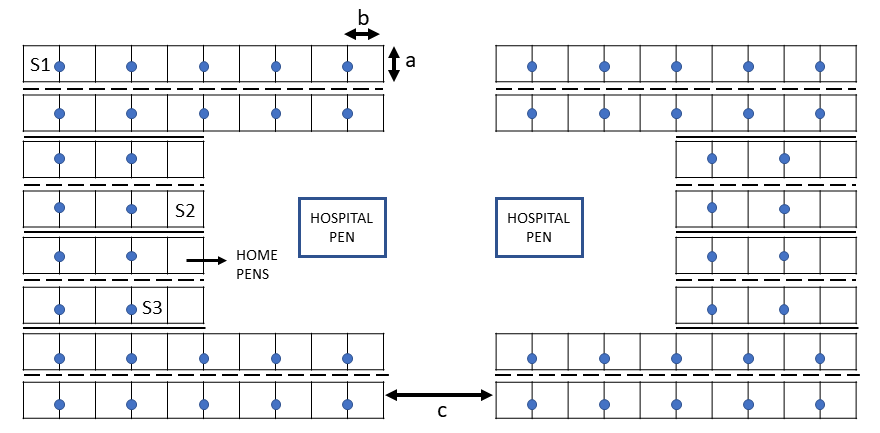** |
| --- |

**Figure S 1D –** FL1 large-size feedlot with two sections and two hospital-pens.

| **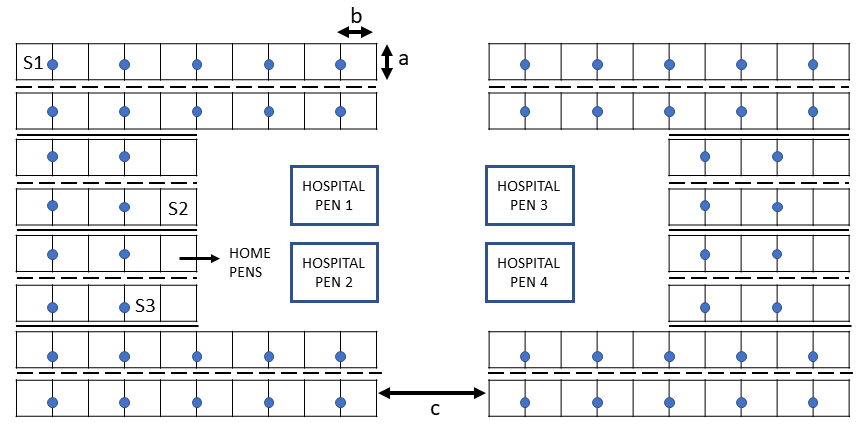** |
| --- |

**Figure S 1E *–*** FL2 large-size feedlot with two sections and four hospital*-*pens
